# Combined statin and fenofibrate therapy and cardiorenal outcomes in patients with CKD, dyslipidemia, and ischemic heart disease

**DOI:** 10.1093/ckj/sfag343

**Published:** 2026-10-01

**Authors:** Yen-Chih Chang, Ming-Hsien Tsai, Yu-Wei Fang, Meng-Ting Chen, Ya-Hui Tseng, Cheng-Chun Wei

**Affiliations:** Division of General Medicine, Department of Internal Medicine, Shin Kong Wu Ho-Su Memorial Hospital, Taipei, Taiwan; Department of Medicine, Fu Jen Catholic University, New Taipei City, Taiwan; Department of Medicine, Fu Jen Catholic University, New Taipei City, Taiwan; Division of Nephrology, Department of Internal Medicine, Shin Kong Wu Ho-Su Memorial Hospital, Taipei, Taiwan; Data Analytic Group, Department of Digital Medicine, Shin Kong Wu Ho-Su Memorial Hospital, Taipei, Taiwan; Department of Medicine, Fu Jen Catholic University, New Taipei City, Taiwan; Division of Nephrology, Department of Internal Medicine, Shin Kong Wu Ho-Su Memorial Hospital, Taipei, Taiwan; Data Analytic Group, Department of Digital Medicine, Shin Kong Wu Ho-Su Memorial Hospital, Taipei, Taiwan; School of Nursing, National Taipei University of Nursing and Health Sciences, Taipei, Taiwan; School of Nursing, National Taipei University of Nursing and Health Sciences, Taipei, Taiwan; Department of Medicine, Fu Jen Catholic University, New Taipei City, Taiwan; Division of Cardiology, Department of Internal Medicine, Shin Kong Wu Ho-Su Memorial Hospital, Taipei, Taiwan

**Keywords:** chronic kidney disease, end-stage kidney disease, hypertriglyceridemia, major adverse cardiovascular event, statin–fenofibrate combination therapy

## Abstract

**Background:**

Adults with non-dialysis-dependent chronic kidney disease (CKD), ischemic heart disease (IHD), and hypertriglyceridemia are at high cardiorenal risk; whether starting fenofibrate together with statin therapy is effective and safe in this population is uncertain.

**Methods:**

In a multicenter retrospective cohort study emulating a target trial in the TriNetX Global Collaborative Network (2018–2024), 112 003 (28.5%) of 392 969 statin initiators with CKD and hypertriglyceridemia had IHD. Those without dialysis dependence were assigned to statin–fenofibrate combination therapy or statin monotherapy according to fenofibrate initiation within 3 months of the first statin prescription. The main outcomes were expanded major adverse cardiovascular events (MACEs) (acute myocardial infarction, stroke, heart failure, cardiac arrest, or all-cause death) and end-stage kidney disease (ESKD) necessitating dialysis. Propensity score matching yielded 3191 pairs. Cox models estimated hazard ratios (HRs).

**Results:**

Median follow-up was 881 days with combination therapy and 925 days with statin monotherapy. Combination therapy was associated with lower risks of MACE [1472 [46.1%] vs 1576 [49.4%] events; HR, 0.93; 95% confidence interval (CI), 0.86–0.99] and ESKD necessitating dialysis (264 [8.3%] vs 335 [10.5%]; HR, 0.79; 95% CI, 0.67–0.92). Risks of all-cause mortality (377 vs. 375), acute myocardial infarction (537 vs. 570), heart failure (1059 vs. 1134), stroke (310 vs. 312), acute kidney injury (825 vs. 840), and coded safety events were not significantly different between groups.

**Conclusions:**

Among selected adults with non-dialysis-dependent CKD, hypertriglyceridemia, and IHD, statin–fenofibrate combination therapy was associated with lower risks of MACE and of ESKD necessitating dialysis, without a significant increase in coded safety events.

## Graphical Abstract

**Graphical Abstract:**
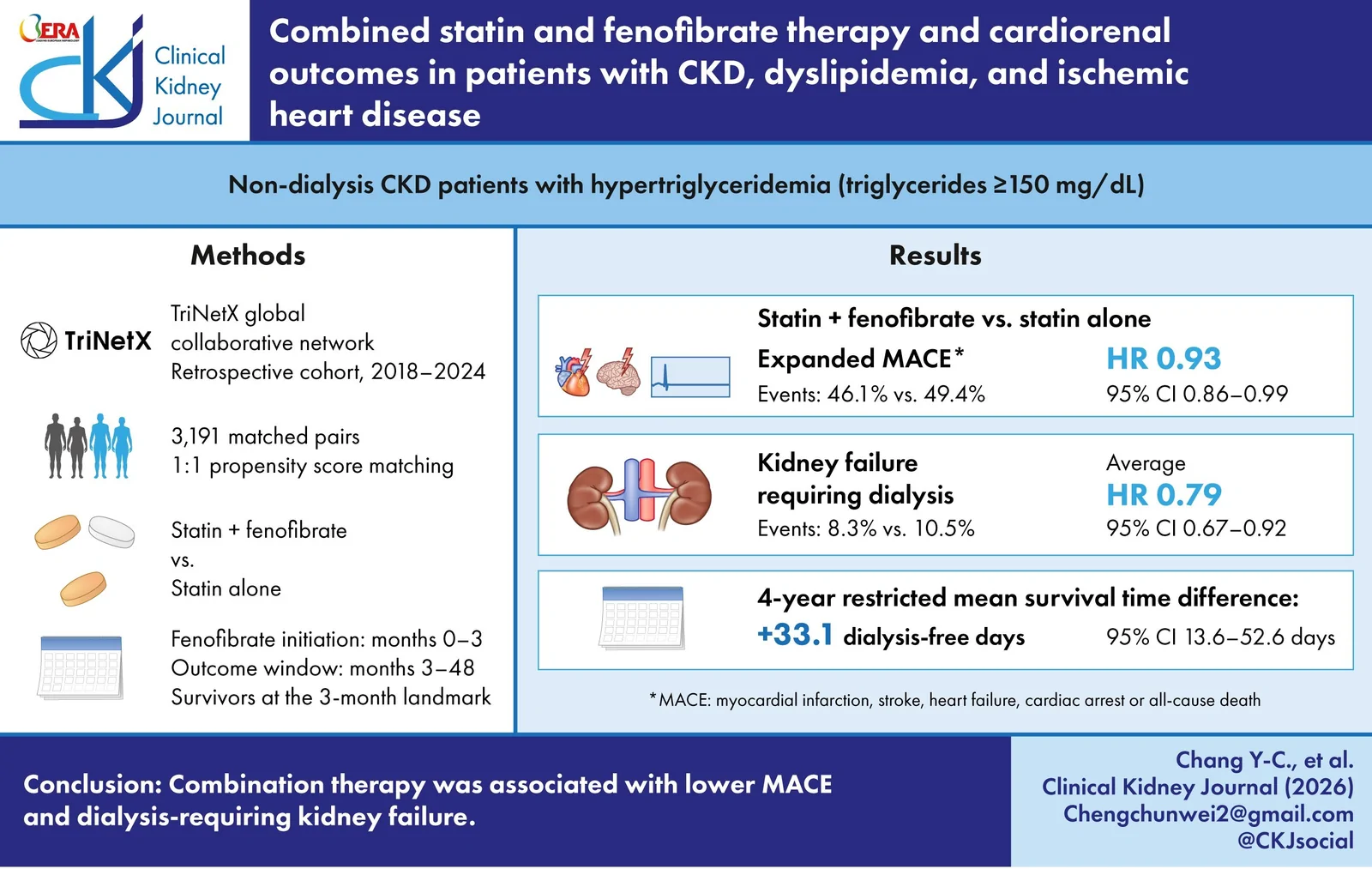

KEY LEARNING POINTS **What was known:**

- Patients with non-dialysis-dependent chronic kidney disease (CKD) and ischemic heart disease remain at high residual cardiorenal risk despite statin therapy.
- Fenofibrate may improve atherogenic dyslipidemia, but concomitant statin–fenofibrate use in CKD remains clinically controversial because of safety concerns.
- Evidence on hard renal outcomes after statin–fenofibrate combination therapy in selected CKD populations has been limited.

**This study adds:**

- In a multicenter emulated target trial, statin–fenofibrate combination therapy was associated with lower risks of major adverse cardiovascular event and dialysis-requiring end-stage kidney disease compared with statin monotherapy.
- Coded rhabdomyolysis, myopathy, liver dysfunction, acute kidney injury, and mortality were not significantly increased in the combination-therapy group.

**Potential impact:**

- These findings may support careful evaluation of statin–fenofibrate therapy in selected non-dialysis-dependent CKD patients with hypertriglyceridemia and ischemic heart disease.
- The results should be interpreted as observational evidence and should not replace individualized renal function monitoring and safety assessment.
- Prospective studies are needed before routine treatment recommendations can be changed.

## INTRODUCTION

Chronic kidney disease (CKD) affected an estimated 697.5 million people worldwide in 2017, corresponding to a global prevalence of 9.1% [1], and is now the seventh leading risk factor for mortality [2]. Cardiovascular disease is a leading cause of death in patients with CKD: approximately half of patients with CKD stages 4–5 have cardiovascular disease, and cardiovascular deaths account for 40%–50% of all deaths in advanced CKD [3]. Ischemic heart disease (IHD) is among the most common and clinically meaningful cardiovascular manifestations in this population [3]. Cases involving both CKD and IHD represent a particularly high-risk phenotype, characterized by accelerated atherosclerosis, endothelial dysfunction, chronic inflammation, oxidative stress, and mineral and lipid metabolism disturbances.

Dyslipidemia is common in patients with CKD and typically manifests as elevated triglyceride levels, reduced high-density lipoprotein cholesterol levels, and altered lipoprotein composition [4, 5]. This atherogenic lipid profile may be particularly relevant in patients with CKD and IHD, in whom residual cardiovascular risk remains high despite standard lipid-lowering therapy. Although statins represent the primary treatment for reducing cardiovascular risk in non-dialysis-dependent CKD, a high residual cardiorenal risk persists, particularly in patients with metabolic comorbidities, atherosclerotic disease, or hypertriglyceridemia [6, 7]. In the placebo group of the REDUCE-IT trial, 22.0% of statin-treated patients with triglyceride levels of 135–499 mg/dl had an ischemic event over a median of 4.9 years [6]. These observations highlight the need for therapeutic strategies beyond conventional statin monotherapy.

Although fenofibrate reduces triglyceride levels, its use in patients with CKD remains a topic of debate [8, 9]. Safety concerns, particularly those related to myopathy or rhabdomyolysis associated with statin–fenofibrate combination therapy, and the commonly observed increase in serum creatinine levels have led to the cautious use of fenofibrate in this population [10–12]. Accordingly, the Kidney Disease: Improving Global Outcomes (KDIGO) lipid guideline recommends avoiding concomitant fibrate and statin use in patients with CKD [13]. However, evidence suggests that the fenofibrate-induced increase in creatinine level is often reversible, particularly in patients with diabetes and hyperlipidemia [14]. In the Fenofibrate Intervention and Event Lowering in Diabetes trial, fenofibrate conferred cardiovascular benefit in patients with an estimated glomerular filtration rate (eGFR) of 30–59 ml/min/1.73 m^2^ without accelerating CKD progression [15]. A 2024 nationwide cohort study reported a reduced incidence of end-stage kidney disease (ESKD) in fenofibrate users, particularly in those with hypertension, proteinuria, or baseline eGFR <60 ml/min/1.73 m^2^ [16]. These findings suggest that fenofibrate use in the CKD population requires careful patient selection and monitoring and that its potential cardiorenal benefit warrants further investigation.

Evidence regarding the cardiorenal outcomes of statin–fenofibrate combination therapy in patients with CKD remains limited. Studies have generally evaluated these agents separately, enrolled select populations that often-excluded patients with advanced CKD, or lacked the power necessary to assess hard outcomes such as ESKD necessitating dialysis. In the present study, we compared cardiorenal outcomes and safety between adults with non-dialysis-dependent CKD, hypertriglyceridemia, and IHD who started fenofibrate within 3 months of statin initiation and those who started statin monotherapy. The objective was to provide real-world evidence on the safety and efficacy of dual lipid-lowering therapy.

## MATERIALS AND METHODS

### Data source

TriNetX is an analytics platform that enables real-world proof-of-concept studies through large, federated networks of healthcare providers by providing access to de-identified electronic health records and, in certain settings, linked claims data [17]. By engaging with the Web-based interface of TriNetX, investigators can define cohorts through explicit eligibility criteria and use built-in tools for feasibility assessments, propensity score matching (PSM), outcome comparison, and data visualization.

### Ethical approval

The study protocol was approved by the Institutional Review Board of Shin Kong Wu Ho-Su Memorial Hospital (approval number: 20260108R). Because TriNetX provides access to a de-identified patient database, the requirement for informed consent was waived.

### Study design and cohort

This retrospective cohort study focused on patients with dyslipidemia and CKD receiving statin therapy. Relevant data were obtained from the TriNetX Global Collaborative Network for 189 765 201 individuals across 105 healthcare organizations from 1 January 2018 to 31 December 2024. Outcome follow-up was available through 6 December 2025. A target trial emulation design was adopted to approximate a randomized controlled trial (Table S1) [18, 19]. This study followed the Strengthening the Reporting of Observational Studies in Epidemiology (STROBE) reporting guideline (Table S2). Using a new-user, active-comparator treatment-initiation design, we identified 484 306 eligible adults who met the following inclusion criteria: having a history of ≥3 visits, being aged ≥18 years, having dyslipidemia, having an eGFR of ≤60 ml/min/1.73 m^2^, having a triglyceride level of ≥150 mg/dl, and using a statin after meeting relevant eligibility criteria. CKD was defined by at least one creatinine-based eGFR ≤60 ml/min/1.73 m^2^ (TriNetX laboratory term labelled as the Modification of Diet in Renal Disease equation), recorded between February 2018 and December 2024 together with a diagnosis of dyslipidemia and a triglyceride level ≥150 mg/dl, before statin initiation; persistence of reduced eGFR and albuminuria were not required. For baseline covariates, all eGFR values recorded during the 1-year baseline window were averaged. We identified 392 969 patients who had not used a statin or fenofibrate within 1 year before the index date—the date of the first statin prescription during the study period. Based on exposure to fenofibrate within 3 months of statin initiation, we assigned 15 452 patients to the dual-therapy group and 377 517 to the statin-monotherapy group. The cohort was then restricted to patients with IHD diagnosed within 1 year before the index date. This restriction resulted in 112 003 patients (28.5% of the 392 969 statin initiators with CKD and hypertriglyceridemia): 5058 in the dual-therapy group and 106 945 in the statin-monotherapy group. Next, prespecified exclusion criteria were applied to the two groups in a single step: patients with any neoplasm or liver disease diagnosed before the index date, those who had undergone dialysis within 6 months before the index date, and those who died within 3 months after the index date were excluded (Fig. 1). Diagnostic and procedural codes are presented in Supplementary Method S1.

**Figure 1:**
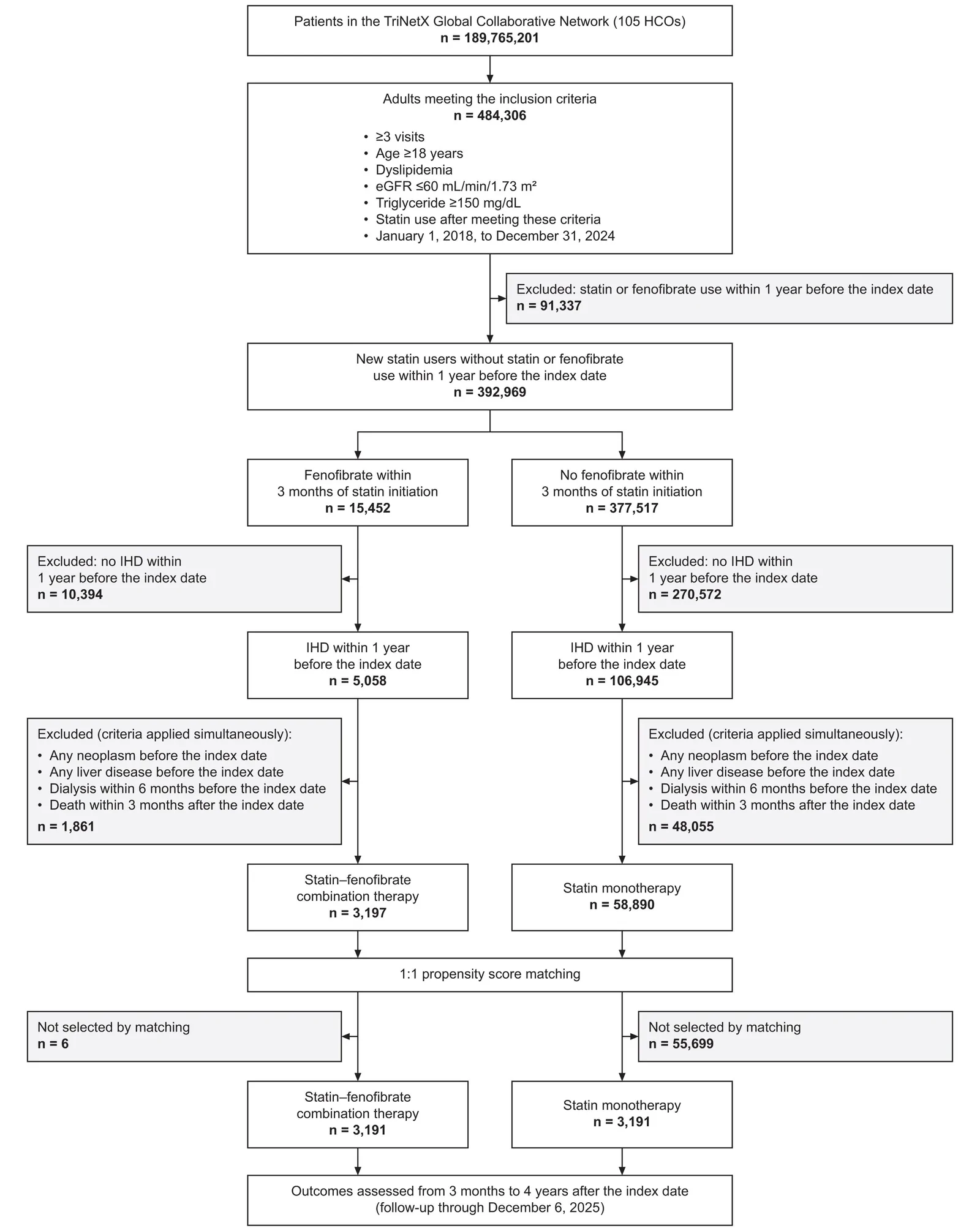
Flowchart of patient selection and PSM. The exclusion criteria in the third step were applied simultaneously; because patients could meet more than one criterion, the number excluded is shown for the step as a whole. Abbreviations: eGFR, estimated glomerular filtration rate; HCO, healthcare organization; IHD, ischemic heart disease.

The final cohort comprised 3197 new recipients of statin–fenofibrate combination therapy and 58 890 new recipients of statin monotherapy. To minimize confounding, 1:1 PSM was performed, thereby balancing baseline covariates between the matched groups. Eligibility and baseline covariates were assessed at statin initiation (time zero). The treatment strategies were initiation of fenofibrate within a 3-month grace period after statin initiation versus no fenofibrate during this period. Because treatment assignment was complete only at the end of the grace period, follow-up started at a 3-month landmark, and patients who died during the grace period were excluded; the estimand is therefore an observational analogue of the intention-to-treat effect among patients who survived the grace period. No adherence rules were applied after assignment (Fig. S1).

### Baseline covariates and PSM

Baseline characteristics were assessed during the year before the index date and included age, sex, body mass index, race, socio-economic and lifestyle factors, healthcare utilization, comorbidities, concomitant medications, and laboratory measures. Health-care utilization comprised outpatient, inpatient, emergency department, and preventive services. Prespecified comorbidities and medications are detailed in Table 1. Laboratory measures included eGFR, triglycerides, and low-density lipoprotein cholesterol. Variables with relatively high availability were prioritized to limit selection bias. Recorded laboratory values were used when available; missing values were not imputed, and missingness was assessed after PSM.

**Table 1:** Baseline characteristics before and after PSM.

	Before matching			After matching		
Characteristic	Statin + fenofibrate (*n* = 3197)	Statin monotherapy (*n* = 58 890)	SMD	Statin + fenofibrate (*n* = 3191)	Statin monotherapy (*n* = 3191)	SMD
Demographic characteristics						
Age, years, mean ± SD	66.4 ± 10.4	67.7 ± 10.9	0.121	66.4 ± 10.4	66.3 ± 11.1	0.015
Male sex, *n* (%)	2223 (69.5)	33 884 (57.5)	0.251	2217 (69.5)	2204 (69.1)	0.009
Race, *n* (%)						
White	2412 (75.4)	41 494 (70.5)	0.112	2412 (75.6)	2429 (76.1)	0.012
Black or African American	140 (4.4)	6501 (11.0)	0.252	140 (4.4)	133 (4.2)	0.011
Asian	70 (2.2)	1947 (3.3)	0.068	70 (2.2)	68 (2.1)	0.004
Other	64 (2.0)	1492 (2.5)	0.036	64 (2.0)	65 (2.0)	0.002
Body mass index, kg/m², mean ± SD	32.2 ± 6.5	31.4 ± 6.8	0.123	32.2 ± 6.5	32.0 ± 6.8	0.022
Socio-economic and lifestyle factors, *n* (%)						
Socio-economic and psychosocial circumstances (Z55⁠–⁠Z65)	71 (2.2)	1587 (2.7)	0.031	71 (2.2)	70 (2.2)	0.002
Health services in other circumstances (Z69⁠–⁠Z76)	448 (14.0)	8769 (14.9)	0.025	448 (14.0)	438 (13.7)	0.009
Family and personal history hazards (Z77⁠–⁠Z99)	2370 (74.1)	43 831 (74.4)	0.007	2368 (74.2)	2386 (74.8)	0.013
Tobacco use	158 (4.9)	3048 (5.2)	0.011	158 (5.0)	154 (4.8)	0.006
Nicotine dependence	529 (16.5)	9535 (16.2)	0.010	529 (16.6)	552 (17.3)	0.019
Alcohol-related disorders	79 (2.5)	1572 (2.7)	0.013	79 (2.5)	93 (2.9)	0.027
Health-care utilization, *n* (%)						
Outpatient services	1334 (41.7)	26 708 (45.4)	0.073	1334 (41.8)	1339 (42.0)	0.003
Inpatient and observation care	1301 (40.7)	23 132 (39.3)	0.029	1301 (40.8)	1318 (41.3)	0.011
Emergency department services	1238 (38.7)	21 981 (37.3)	0.029	1238 (38.8)	1232 (38.6)	0.004
Preventive medicine services	124 (3.9)	2130 (3.6)	0.014	124 (3.9)	112 (3.5)	0.020
Comorbidities, *n* (%)						
Hypertensive diseases	2933 (91.7)	53 459 (90.8)	0.034	2927 (91.7)	2946 (92.3)	0.022
Diabetes mellitus	2404 (75.2)	35 361 (60.0)	0.328	2398 (75.1)	2417 (75.7)	0.014
Heart failure	1055 (33.0)	21 069 (35.8)	0.058	1055 (33.1)	1079 (33.8)	0.016
Atrial fibrillation and flutter	635 (19.9)	12 370 (21.0)	0.028	635 (19.9)	640 (20.1)	0.004
Other cardiac arrhythmias	375 (11.7)	8037 (13.6)	0.058	375 (11.8)	368 (11.5)	0.007
Cardiomyopathy	263 (8.2)	5785 (9.8)	0.056	263 (8.2)	273 (8.6)	0.011
Cerebrovascular diseases	607 (19.0)	12 506 (21.2)	0.056	607 (19.0)	628 (19.7)	0.017
Chronic lower respiratory diseases	779 (24.4)	13 671 (23.2)	0.027	779 (24.4)	802 (25.1)	0.017
Chronic obstructive pulmonary disease	595 (18.6)	9451 (16.0)	0.068	595 (18.6)	617 (19.3)	0.018
Disorders of thyroid gland	671 (21.0)	11 260 (19.1)	0.047	671 (21.0)	711 (22.3)	0.030
Disorders of mineral metabolism	344 (10.8)	6715 (11.4)	0.020	344 (10.8)	365 (11.4)	0.021
Gout	328 (10.3)	5692 (9.7)	0.020	326 (10.2)	327 (10.2)	0.001
Anxiety disorders	582 (18.2)	9970 (16.9)	0.034	580 (18.2)	606 (19.0)	0.021
Polyneuropathy	153 (4.8)	3050 (5.2)	0.018	153 (4.8)	157 (4.9)	0.006
Dementia	34 (1.1)	709 (1.2)	0.013	34 (1.1)	39 (1.2)	0.015
Medications, *n* (%)						
Antithrombotic agents	2740 (85.7)	47 268 (80.3)	0.145	2734 (85.7)	2741 (85.9)	0.006
Beta-blockers	2551 (79.8)	43 081 (73.2)	0.157	2545 (79.8)	2551 (79.9)	0.005
Renin–angiotensin system blockers	1935 (60.5)	31 543 (53.6)	0.141	1929 (60.5)	1948 (61.0)	0.012
Insulin	1805 (56.5)	26 372 (44.8)	0.235	1800 (56.4)	1806 (56.6)	0.004
Diuretics	1611 (50.4)	28 223 (47.9)	0.049	1609 (50.4)	1628 (51.0)	0.012
Calcium channel blockers	1518 (47.5)	26 451 (44.9)	0.051	1514 (47.4)	1555 (48.7)	0.026
Proton pump inhibitors	1335 (41.8)	22 197 (37.7)	0.083	1333 (41.8)	1370 (42.9)	0.023
Antiarrhythmics	1330 (41.6)	25 637 (43.5)	0.039	1330 (41.7)	1351 (42.3)	0.013
Oral hypoglycemic agents	1254 (39.2)	15 445 (26.2)	0.280	1248 (39.1)	1234 (38.7)	0.009
Thyroid therapy	616 (19.3)	8422 (14.3)	0.133	614 (19.2)	661 (20.7)	0.037
Nonsteroidal anti-inflammatory drugs	521 (16.3)	9801 (16.6)	0.009	521 (16.3)	529 (16.6)	0.007
Aldosterone antagonists	283 (8.9)	4756 (8.1)	0.028	283 (8.9)	273 (8.6)	0.011
Allopurinol	281 (8.8)	4517 (7.7)	0.041	281 (8.8)	288 (9.0)	0.008
Peripheral vasodilators	178 (5.6)	1540 (2.6)	0.149	176 (5.5)	186 (5.8)	0.014
Febuxostat	79 (2.5)	896 (1.5)	0.068	77 (2.4)	80 (2.5)	0.006
Laboratory values						
eGFR, ml/min/1.73 m², mean ± SD	47.9 ± 21.4	48.2 ± 23.2	0.012	47.9 ± 21.4	47.4 ± 23.3	0.024
Triglycerides, mg/dl, mean ± SD	315.6 ± 282.4	221.7 ± 127.9	0.428	315.2 ± 282.5	289.6 ± 220.8	0.101
≥500 mg/dl, *n* (%)	443 (13.9)	1913 (3.2)	0.386	437 (13.7)	407 (12.8)	0.028
LDL cholesterol, mg/dl, mean ± SD	81.8 ± 39.5	93.2 ± 45.7	0.268	81.8 ± 39.5	82.9 ± 40.3	0.027
≥190 mg/dl, *n* (%)	57 (1.8)	2030 (3.4)	0.104	57 (1.8)	60 (1.9)	0.007

Values are *n* (%) or mean ± SD. An SMD <0.1 indicates adequate balance between groups.

Laboratory values and body mass index are the mean of all measurements in the year before the index date, among patients with at least one measurement (after matching: eGFR, 97.8% and 98.4%; triglycerides, 92.0% and 92.3%; LDL cholesterol, 83.1% and 84.4%; body mass index, 66.3% and 66.9%). Categories are based on all patients in each group. Individual glucose-lowering drug classes before and after matching are reported in Table S6. Abbreviations: eGFR, estimated glomerular filtration rate; LDL, low-density lipoprotein; SD, standard deviation; SMD, standardized mean difference.

Propensity scores were calculated using logistic regression, and 1:1 PSM was performed on the TriNetX platform (Supplementary Method S2) by using a greedy nearest-neighbor algorithm with a caliper of 0.1 pooled standard deviations of the propensity score. This approach has been demonstrated to enhance the comparability of treatment groups, with a standardized mean difference (SMD) of <0.1 indicating a negligible imbalance [20, 21]. During PSM, age on the index date was treated as a continuous variable, whereas sex, race, lifestyle-related factors, comorbidities, and medication use were coded as binary categorical variables. Laboratory parameters were classified as present if they were recorded within the specified time frame or as absent if they were missing. The two groups exhibited similar rates of missing data after PSM.

### Study outcomes

The main outcomes were expanded major adverse cardiovascular events (MACEs), defined as the first occurrence of acute myocardial infarction, stroke, heart failure, cardiac arrest, or all-cause death, and ESKD necessitating dialysis. Secondary outcomes were all-cause death, acute myocardial infarction, heart failure, stroke, and acute kidney injury. Outcomes occurring within 3–48 months after the index date were examined. Patient follow-up and data collection continued throughout the study period and concluded on 6 December 2025. For non-fatal outcomes, patients who died or were lost to follow-up during the observation period were treated as censored at the date of death or last recorded encounter. Safety outcomes (rhabdomyolysis, impaired liver function, myopathy, and functional diarrhea) and an alternative definition of ESKD based on diagnosis codes indicating chronic kidney failure (N18.6 or Z99.2) were assessed in a separate matched analysis using the same cohort definitions.

### Sensitivity analysis

Several prespecified sensitivity analyses were conducted to assess the robustness of the findings. First, negative control outcomes, including accidental falls, pressure ulcers, and screening colonoscopies, were analyzed to evaluate potential residual confounding and analytical bias. Second, primary outcome models were re-estimated using three progressively inclusive propensity score specifications—baseline demographic characteristics and clinical measures, followed by sequential addition of comorbidities and concomitant medications. In an additional analysis, the propensity score model was extended by replacing oral hypoglycemic agents with seven drug classes [sodium–glucose co-transporter 2 (SGLT2) inhibitors, glucagon-like peptide-1 (GLP-1) receptor agonists, dipeptidyl peptidase-4 inhibitors, biguanides, sulfonylureas, alpha-glucosidase inhibitors, and thiazolidinediones] and adding acute kidney injury, glycated hemoglobin (HbA1c), systolic and diastolic blood pressure, and categories of eGFR (≤15, 15–30, and 30–60 ml/min/1.73 m^2^), HbA1c, and body mass index. Third, to examine whether the associations changed with the length of follow-up, the Cox models were re-estimated in four cumulative follow-up windows, each starting at the 3-month landmark and ending at 1, 2, 3, and 5 years after the index date, with administrative censoring at the end of each window. In addition, the target trial was emulated without a grace period in a separately matched cohort, in which combination therapy was defined as fenofibrate prescribed on the same day as the first statin prescription, the comparator was statin initiation without fenofibrate on that day, and follow-up ran from the day after the index date to 4 years without exclusion of early deaths. Finally, *E*-values were calculated to quantify the minimum magnitude of unmeasured confounding necessary to nullify the observed treatment associations [22].

### Statistical analysis

Categorical variables are presented as counts and percentages, and continuous variables are presented as mean ± standard deviation values. SMDs were used to establish comparable groups by minimizing baseline confounding; an SMD of <0.1 indicated a good balance in demographic, clinical, and laboratory characteristics at baseline [21]. Cox proportional hazards regression was used to estimate hazard ratios (HRs) and the cardiorenal risks associated with statin–fenofibrate combination therapy and statin monotherapy. The proportional hazards assumption was verified using the generalized Schoenfeld method in TriNetX. When the assumption was not met, the HR was interpreted as an average HR over follow-up. Kaplan–Meier survival curves and log-rank tests were used to compare event-free survival between the two groups.

To augment relative risk estimates, absolute effect measures were calculated. Absolute risk reduction (ARR) was defined as the difference in cumulative incidence between the treatment groups over the follow-up period. The number needed to treat (NNT) was calculated as the inverse of ARR. NNT reflected the number of patients requiring combination therapy to prevent an additional event. Restricted mean survival time differences (RMSTDs) were also estimated as the difference in area under the survival curve up to the predefined follow-up horizon, which represents the average event-free time gained without the assumption of proportional hazards [23, 24]. ARRs, NNTs, and RMSTDs were used to translate the relative risk estimates into clinically meaningful absolute effects. Because the proportional hazards assumption was not met for ESKD necessitating dialysis, the RMSTD was used as the principal renal estimate.

Prespecified subgroup analyses were conducted to investigate the associations between statin–fenofibrate combination therapy (vs. statin monotherapy) and cardiorenal outcomes. Because TriNetX does not support interaction terms, subgroup-specific HRs were estimated in separately matched subgroups, and differences between them were assessed with Cochran’s *Q* test, which for two independent estimates is equivalent to testing the difference between the log HRs [25]. This is not a model-based test of treatment-by-subgroup interaction, and the 26 comparisons (13 subgroup variables for 2 outcomes) were not adjusted for multiplicity. PSM was applied for all baseline variables in each subgroup to ensure well balanced and comparable pairs.

No data were imputed. TriNetX performed PSM using Python and R version 3.4.4. Two-sided *P* values <0.05 denoted statistical significance. Figures were drawn with matplotlib 3.10 in Python 3.14. Because TriNetX does not export numbers at risk, these were back-calculated with custom Python scripts from the exported Kaplan–Meier estimates and the Greenwood variance underlying their 95% confidence intervals (CIs), analogous to the reconstruction of survival data from published Kaplan–Meier curves [26]. The back-calculated numbers reproduced the matched cohort size (3191 per group) and the event counts reported by the platform.

## RESULTS

### Baseline characteristics

Before PSM, 3197 patients received combination therapy and 58 890 received statin monotherapy (Table 1). Combination-therapy recipients were younger (66.4 vs. 67.7 years), more often male (69.5% vs. 57.5%), and had higher body mass index (32.2 vs. 31.4 kg/m^2^) and triglyceride levels (315.6 vs. 221.7 mg/dl). They were also more likely to have diabetes and to receive insulin, oral hypoglycemic agents, beta-blockers, antithrombotic agents, renin–angiotensin system blockers, and thyroid therapy. They were less often African American and had lower low-density lipoprotein cholesterol levels. Baseline renal function was similar.

After 1:1 PSM, each group included 3191 patients. Baseline covariates were well balanced, with all SMDs <0.1 except triglyceride levels (SMD, 0.101). Mean follow-up was 862 and 883 days, and median follow-up was 881 and 925 days, in the combination-therapy and statin-monotherapy groups, respectively (Fig. S2).

### Cardiorenal outcomes

Kaplan–Meier analysis showed higher event-free survival for MACE and ESKD necessitating dialysis with combination therapy over 4 years (Fig. 2). Log-rank *P* values were .034 for MACE and .003 for ESKD. No significant differences were observed for the other outcomes (Fig. S3).

**Figure 2:**
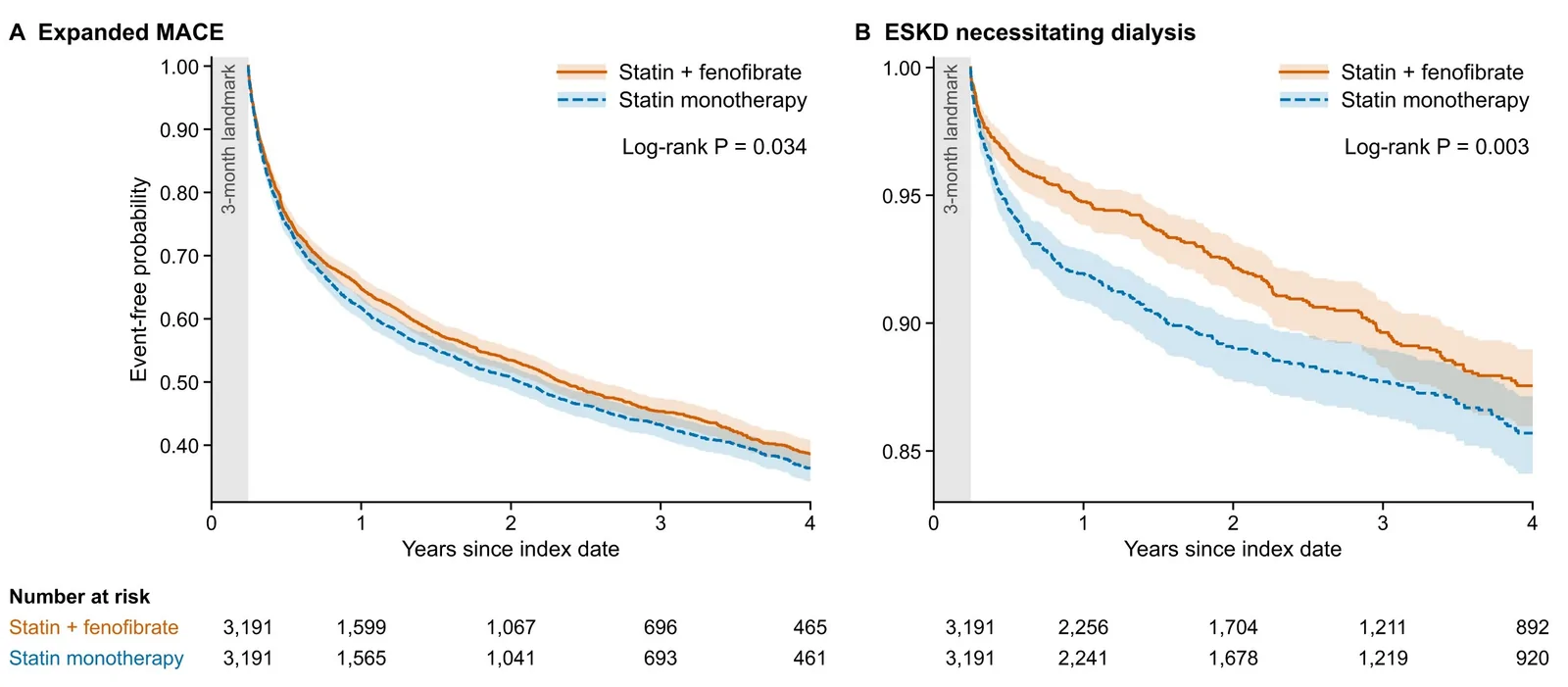
Kaplan–Meier curves for (A) MACE and (B) ESKD necessitating dialysis in the propensity score-matched cohort. The grey area marks the first 3 months after the index date; follow-up began at this landmark, and patients who died during this interval were excluded. Colored bands indicate 95% confidence intervals; *P* values are from log-rank tests. Numbers at risk, back-calculated from the Kaplan–Meier estimates exported by TriNetX, are shown below each panel. Abbreviations: ESKD, end-stage kidney disease; MACE, expanded major adverse cardiovascular events.

Dual therapy was associated with a lower risk of MACE (46.1% vs. 49.4%; HR: 0.93, 95% CI 0.86–0.99) (Table 2). For ESKD necessitating dialysis, 8.3% vs. 10.5% of patients had an event over 4 years, and the RMSTD at 4 years was 33.1 days (95% CI: 13.6–52.6); the average HR was 0.79 (95% CI 0.67–0.92). The proportional hazards assumption was met for MACE (*P* = .413) but not for ESKD necessitating dialysis (*P* = .003). The corresponding *E*-values were 1.36 and 1.85, respectively. No significant between-group differences were observed in the other outcomes.

**Table 2:** Cardiorenal outcomes in the propensity score-matched cohort.

	Statin + fenofibrate (*n* = 3191)	Statin monotherapy (*n* = 3191)	Statin + fenofibrate vs. statin monotherapy		
Outcome	Events, *n* (%)	Events, *n* (%)	HR (95% CI)^a^	*P* value^b^	*E*-value^c^
Main outcomes					
Expanded MACE^d^	1472 (46.1)	1576 (49.4)	0.93 (0.86–0.99)	.034	1.36 (1.11)
ESKD necessitating dialysis^e^	264 (8.3)	335 (10.5)	0.79 (0.67–0.92)	.003	1.85 (1.39)
Secondary outcomes					
All-cause mortality	377 (11.8)	375 (11.8)	1.03 (0.89–1.19)	.684	1.21 (1.00)
Acute myocardial infarction	537 (16.8)	570 (17.9)	0.96 (0.85–1.08)	.474	1.25 (1.00)
Heart failure	1059 (33.2)	1134 (35.5)	0.94 (0.86–1.02)	.119	1.32 (1.00)
Stroke	310 (9.7)	312 (9.8)	1.02 (0.87–1.19)	.825	1.16 (1.00)
Acute kidney injury	825 (25.9)	840 (26.3)	1.01 (0.92–1.11)	.864	1.11 (1.00)

a Cox proportional hazards models; follow-up from 3 months to 4 years after the index date.

b Log-rank test.

c *E*-value for the point estimate, with the *E*-value for the confidence limit closest to the null in parentheses.

d Acute myocardial infarction, stroke, heart failure, cardiac arrest, or all-cause death.

e The proportional hazards assumption was not met for this outcome (Schoenfeld test, *P* = .003); the HR is an average over follow-up.

Abbreviations: CI, confidence interval; ESKD, end-stage kidney disease; HR, hazard ratio; MACE, major adverse cardiovascular event.

### Clinical application

Table S3 presents the differential effects of statin–fenofibrate combination therapy. For MACE, the ARR was greatest in the first 2 years: 30.7 events prevented per 1000 patients at 1 year (NNT at 1 year, 33; 95% CI 18–188) and 28.8 per 1000 patients at 2 years (NNT at 2 years, 35; 95% CI 18–702). The ARRs at 3 and 4 years (21.4 and 22.3 events prevented per 1000 patients) were not statistically significant.

For ESKD necessitating dialysis, the ARR was significant through 3 years, with 28.1, 31.7, and 19.3 events prevented per 1000 patients at 1, 2, and 3 years (NNT at 1 year, 36 [95% CI 25–67]; at 2 years, 32 [21–65]; at 3 years, 52 [27–1195]); the estimate at 4 years was not statistically significant.

### Results of subgroup analysis

Subgroup HRs generally favored combination therapy for MACE and ESKD necessitating dialysis (Figs 3 and 4). The difference between subgroups was nominally significant only for age in the MACE analysis (≥65 years: HR 0.87, 95% CI 0.80–0.96; 18–64 years: HR 1.06, 95% CI 0.94–1.19; *P* = .009), which was not significant after Bonferroni correction for 26 comparisons.

**Figure 3:**
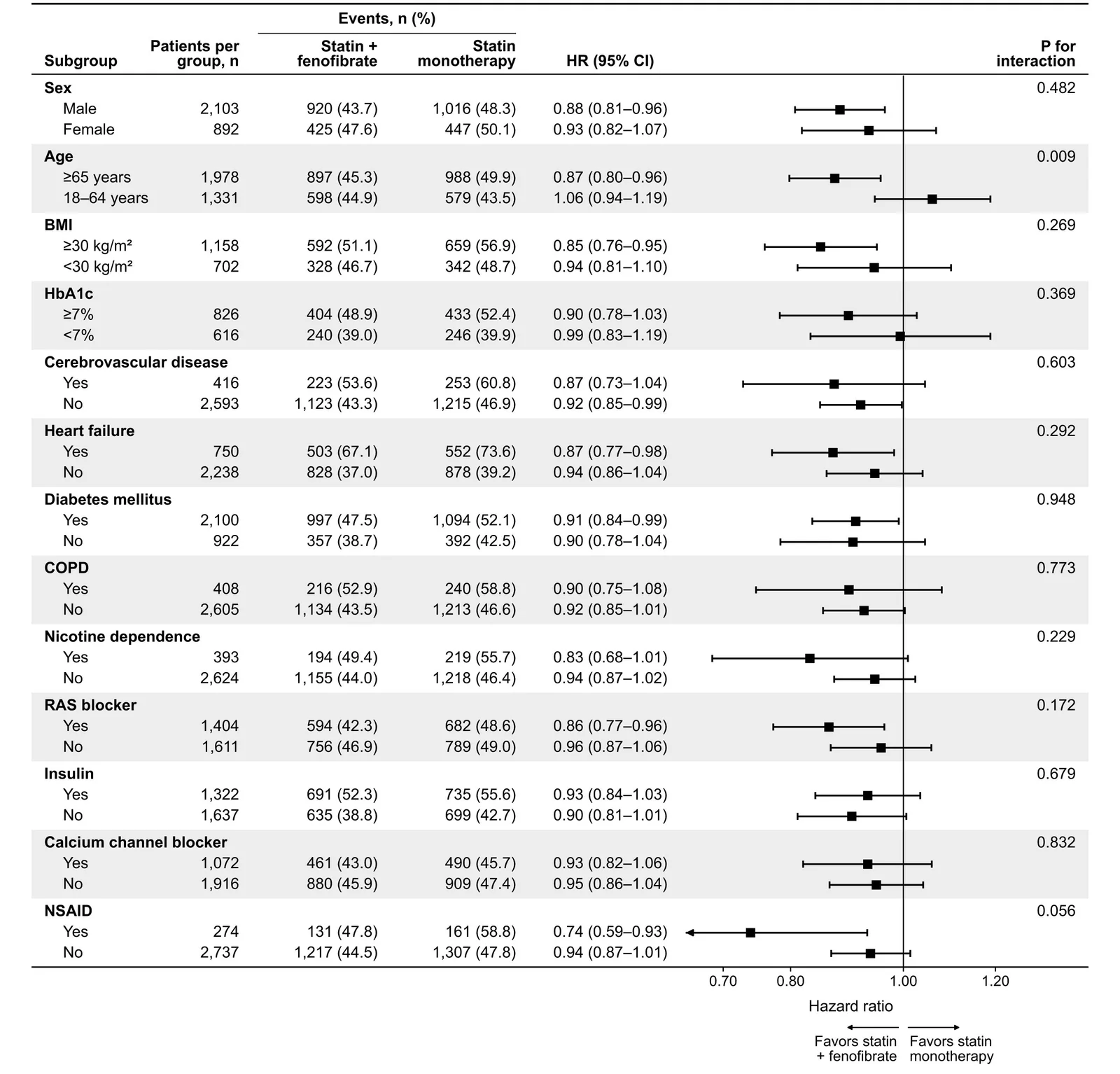
Subgroup analyses of expanded MACE. For each subgroup, the numbers of patients and events in each group and the HR with 95% CI are shown. *P* for interaction is from Cochran’s *Q* test for the difference between subgroup-specific HRs; 26 comparisons were made across Figs 3 and 4, and none was statistically significant after Bonferroni correction (threshold *P* < .002). Abbreviations: BMI, body mass index; CI, confidence interval; COPD, chronic obstructive pulmonary disease; HbA1c, glycated hemoglobin; HR, hazard ratio; MACE, major adverse cardiovascular event; NSAID, nonsteroidal anti-inflammatory drug; RAS, renin–angiotensin system.

**Figure 4:**
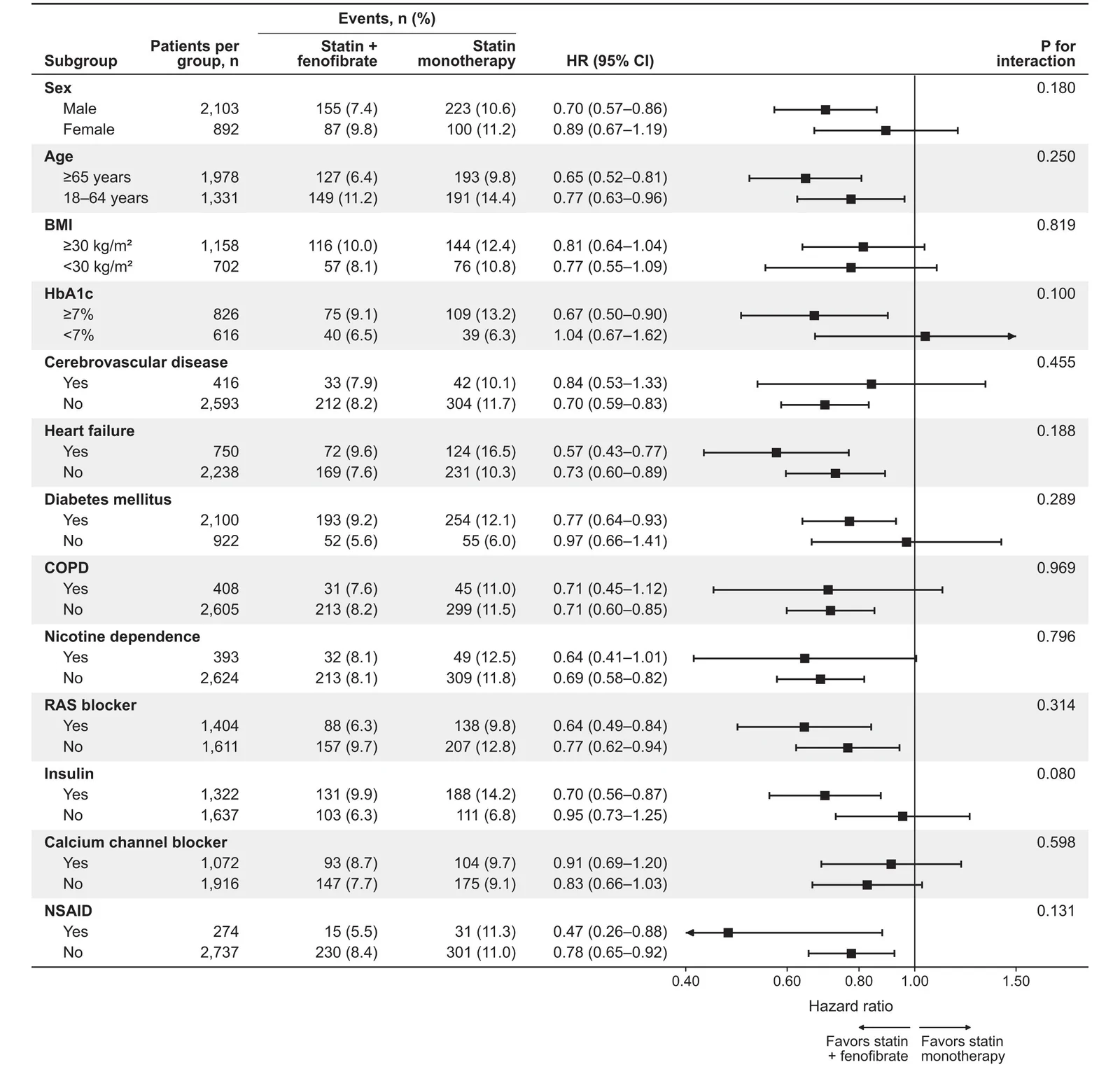
Subgroup analyses of ESKD necessitating dialysis. For each subgroup, the numbers of patients and events in each group and the HR with 95% CI are shown. *P* for interaction is from Cochran’s *Q* test for the difference between subgroup-specific HRs; 26 comparisons were made across Figs 3 and 4, and none was statistically significant after Bonferroni correction (threshold *P* < .002). Abbreviations: BMI, body mass index; CI, confidence interval; COPD, chronic obstructive pulmonary disease; ESKD, end-stage kidney disease; HbA1c, glycated hemoglobin; HR, hazard ratio; NSAID, nonsteroidal anti-inflammatory drug; RAS, renin–angiotensin system.

### Results of sensitivity analysis

Falsification tests using negative control outcomes—accidental falls, pressure ulcers, and screening colonoscopies—indicated no significant differences (Table S4). Progressive propensity score adjustment models revealed that the ESKD associations became stronger with more extensive adjustment (Model 1, HR: 0.77, *P* = .001; Model 3, HR: 0.74, *P* < .001), whereas MACE associations were attenuated (Model 1, HR: 0.85, *P* < .001; Model 3, HR: 0.97, *P* = .333) (Table S5). In the extended propensity score model (3644 matched pairs) (Table S6), the HRs were 0.89 (95% CI 0.84–0.95) for MACE and 0.84 (95% CI 0.72–0.97) for ESKD necessitating dialysis (Table S7). Across all follow-up windows (3 months to 5 years), HRs ranged from 0.73 to 0.77 for ESKD necessitating dialysis and from 0.91 to 0.93 for MACE (Table S8). When combination therapy was defined as same-day co-prescription and follow-up started on the day after the index date (2175 matched pairs), the HRs were 0.84 (95% CI 0.78–0.91) for MACE and 0.74 (95% CI 0.62–0.89) for ESKD necessitating dialysis (Table S9). With ESKD defined by diagnosis codes for chronic kidney failure (N18.6 or Z99.2), the HR was 0.80 (95% CI 0.69–0.93; 3660 matched pairs) (Table S10).

### Safety profile

Safety outcomes were assessed in a separate matched analysis with the same cohort definitions (3660 pairs) (Table S10). Rhabdomyolysis occurred in 41 (1.1%) patients in the combination-therapy group and 31 (0.8%) in the statin-monotherapy group (HR 1.34, 95% CI 0.84–2.14), impaired liver function in 192 (5.2%) and 209 (5.7%) (HR 0.92, 95% CI 0.76–1.12), and myopathy in 44 (1.2%) and 37 (1.0%) (HR 1.21, 95% CI 0.78–1.87). The number of patients with functional diarrhea was too small for results to be reported by the platform.

## DISCUSSION

In this multicenter emulated target trial study of adults with non-dialysis-dependent CKD, IHD, and hypertriglyceridemia, starting fenofibrate within 3 months of statin initiation was associated with lower risks of MACE and ESKD necessitating dialysis compared with statin monotherapy. These associations were observed without a statistically significant increase in coded rhabdomyolysis, myopathy, liver dysfunction, acute kidney injury, or mortality. These findings suggest that co-initiating fenofibrate with a statin may confer cardiorenal benefit in selected patients with CKD and hypertriglyceridemia initiating statin therapy, with a clinically meaningful absolute effect: at 2 years, ∼29 MACE events and 32 dialysis-requiring ESKD events were prevented per 1000 patients treated.

These results extend prior evidence on the mixed cardiorenal effects of fibrates in CKD. The KDIGO lipid guideline has historically discouraged concomitant statin and fibrate use in CKD because of toxicity concerns [13], whereas randomized and observational data suggest that fenofibrate-associated creatinine increases may be reversible and that fenofibrate may reduce albuminuria or delay kidney disease progression in selected populations [14–16, 27]. By focusing on dialysis initiation rather than creatinine-based outcomes, the present study assessed a hard renal endpoint that is less vulnerable to transient changes in creatinine handling [28].

Fenofibrate activates peroxisome proliferator-activated receptor-alpha, improves triglyceride-rich lipoprotein metabolism, and may attenuate inflammatory, endothelial, and microvascular pathways relevant to CKD progression [29]. The renal association was consistent across sensitivity analyses.

In contrast, the cardiovascular association was modest and less consistent across propensity score specifications (Tables S5 and S7). This pattern is consistent with prior trial and cohort findings showing heterogeneous cardiovascular effects of fenofibrate, particularly when added to statin therapy [30, 31]. Heart failure, the most frequent component of MACE, showed a directionally consistent association (HR 0.94; 95% CI 0.86–1.02) that reached statistical significance within the first 2 years of follow-up (HR 0.90; 95% CI 0.82–0.99; Table S8); whether fenofibrate reduces heart failure events in this population warrants confirmation in larger studies. SGLT2 inhibitors reduce kidney and cardiovascular events in patients with CKD [3], and the use of GLP-1 receptor agonists and thiazolidinediones is shaped by kidney function and cardiac status; differences in these therapies could therefore influence cardiorenal outcomes. In the extended propensity score model, these drug classes were balanced between groups, and the associations were similar to those of the primary analysis.

Safety remains central to clinical interpretation, as statin–fibrate combination therapy has been associated with a higher incidence of hospitalized rhabdomyolysis than statin monotherapy [32]. In the present cohort, coded rhabdomyolysis, myopathy, and impaired liver function were not significantly more frequent with combination therapy. These findings should not be extrapolated to dialysis populations, transplant recipients, advanced CKD patients in whom fenofibrate is contraindicated, or patients without careful renal and safety monitoring.

This study has several strengths, including a large multicenter real-world database, a new-user active-comparator framework, target trial emulation, extensive PSM across demographic characteristics, comorbidities, medications, healthcare utilization, laboratory measures, and prespecified sensitivity and negative control analyses. Nevertheless, limitations include residual confounding, including confounding by indication from preferential prescribing of fenofibrate to patients with stable kidney function and unmeasured pre-index eGFR trajectories (an unmeasured confounder would need to be associated with both treatment and outcome by a risk ratio of at least 1.36 to explain away the MACE association and 1.85 for ESKD). Because treatment was classified during a 3-month grace period, the primary estimand is conditional on survival to the landmark; cloning–censoring–weighting approaches, which avoid this conditioning, require patient-level data that are not available on the federated platform. In addition, exposure was based on prescription records, and information on dispensing, adherence, persistence, statin intensity, and fenofibrate dose (including renal dose adjustment) was not available; discontinuation in the combination group or later fenofibrate use in the monotherapy group would tend to bias the comparison towards the null; and post-baseline eGFR and lipid levels, measured only when clinically indicated, could not be analyzed as trajectories.

CKD was defined by a single eGFR measurement without confirmation of chronicity, and albuminuria could not be assessed because the urine albumin-to-creatinine ratio was rarely measured in routine care. Dialysis-requiring ESKD was identified from procedure and diagnosis codes without a validated kidney-failure algorithm, heart failure events were identified from diagnosis codes and could not be distinguished from encounters for prevalent heart failure, and, because competing-risk models are not available on the platform, death was treated as a censoring event for non-fatal outcomes, so that Kaplan–Meier estimates overstate their absolute cumulative incidence. Muscle and hepatic adverse events during the 3-month grace period, creatinine elevation, and drug intolerance could not be assessed, and power was limited for rare safety outcomes. Subgroup analyses had limited power, and because Cochran’s *Q* is not a model-based test of treatment-by-subgroup interaction, differences in statistical significance between subgroups should not be interpreted as effect modification. Finally, generalizability is restricted to TriNetX patients with non-dialysis-dependent CKD, IHD, hypertriglyceridemia, and sufficient healthcare engagement.

In conclusion, among selected adults with non-dialysis-dependent CKD, IHD, and hypertriglyceridemia initiating statin therapy, co-initiation of fenofibrate was associated with a lower 4-year risk of dialysis-requiring ESKD and a modestly lower risk of MACE compared with statin monotherapy. Prospective trials are warranted to establish whether fenofibrate slows progression to kidney failure in this population.

## Supplementary Material

sfag343_Supplemental_File

## Data Availability

The de-identified electronic health record data supporting this study are available through the TriNetX platform and are not publicly downloadable because they are contributed by participating health-care organizations under data-use and privacy restrictions. Qualified researchers may request access through TriNetX subject to institutional approval, applicable agreements, and platform permissions. No custom analytic code beyond standard TriNetX functions and the Python scripts used for figure generation and for reconstructing numbers at risk is publicly deposited.
