# Supplementary material for: Combined statin and fenofibrate therapy and cardiorenal outcomes in patients with CKD, dyslipidemia, and ischemic heart disease
Source: Clin Kidney J. 2026 Oct 1;19(10):sfag343. doi: 10.1093/ckj/sfag343 (PMC13655736; doi:10.1093/ckj/sfag343)
Supplement: Supplementary file 1 [file sfag343_supplemental_file.docx]

**Supplementary Online Content**

Method S1. Codes for cohorts, outcomes, and baseline data	2

1. Codes for cohorts	2

2. Codes for outcome definitions	2

3. Codes for the baseline covariates for propensity score matching	4

Method S2. The details of propensity score matching in the TriNetX platform	6

Figure S1. Schematic of study design	13

Figure S2. Distribution of follow-up time in the propensity score–matched cohort	14

Figure S3. Kaplan–Meier curves for secondary outcomes in the propensity score–matched cohort	15

Table S1. Target trial emulation	16

Table S2. STROBE statement	19

Table S3. Annual absolute risk reduction and clinical impact	23

Table S4. Falsification test	24

Table S5. Propensity score matching models	25

Table S6. Baseline characteristics with the extended covariate set	26

Table S7. Clinical outcomes with the extended covariate set	28

Table S8. Hazard ratios over cumulative follow-up windows starting at the 3-month landmark	29

Table S9. Clinical outcomes with combination therapy defined as same-day co-prescription	30

Table S10. Alternative definition of ESKD and safety outcomes in a separate matched analysis	31

# **Method S1. Codes for cohorts, outcomes, and baseline data**

## 1. **Codes for cohorts**

| **Type** | **Code** | **Description** |
| --- | --- | --- |
| **Inclusion criteria** | | |
| **Lipid disorders, eGFR, triglycerides, ischemic heart disease, and study medications** | | |
| Diagnosis | UMLS:ICD10CM:E78 | Disorders of lipoprotein metabolism and other lipidemias (at least 18 years old at event) |
| Laboratory | TNX:8001 | Glomerular filtration rate/1.73 sq M.predicted [Volume Rate/Area] in Serum, Plasma or Blood by Creatinine-based formula (MDRD) (at most 60.00 mL/min/1.73 m²) |
| Laboratory | TNX:9004 | Triglyceride [Mass/volume] in Serum, Plasma or Blood (at least 150.00 mg/dL) |
| Medication | NLM:ATC:C10AA | HMG CoA reductase inhibitors |
| Medication | NLM:RXNORM:8703 | Fenofibrate |
| Diagnosis | UMLS:ICD10CM:I20-I25 | Ischemic heart diseases (within 1 year before the index date) |
| **Visit** | | |
| Visit | TNX:Visit | Visit |
| **Exclusion criteria** | | |
| **Neoplasms, liver disease, dialysis, and death** | | |
| Diagnosis | UMLS:ICD10CM:C00-D49 | Neoplasms |
| Diagnosis | UMLS:ICD10CM:K70-K77 | Diseases of liver |
| Procedure | UMLS:CPT:90945 | Dialysis procedure other than hemodialysis (eg, peritoneal dialysis, hemofiltration, or other continuous renal replacement therapies), with single evaluation by a physician or other qualified health care professional |
| Procedure | UMLS:CPT:1012740 | Dialysis Services and Procedures |
| Demographics | Deceased | Deceased |

## 2. **Codes for outcome definitions**

The table below outlines the definitions for each outcome and the analysis specifications. For outcome definitions consisting of more than one term, at least one term must match.

| **Type** | **Code** | **Description** |
| --- | --- | --- |
| **Main outcomes** | | |
| **Expanded MACE** | | |
| Diagnosis | UMLS:ICD10CM:I21 | Acute myocardial infarction |
| Diagnosis | UMLS:ICD10CM:I60 | Nontraumatic subarachnoid hemorrhage |
| Diagnosis | UMLS:ICD10CM:I61 | Nontraumatic intracerebral hemorrhage |
| Diagnosis | UMLS:ICD10CM:I62 | Other and unspecified nontraumatic intracranial hemorrhage |
| Diagnosis | UMLS:ICD10CM:I63 | Cerebral infarction |
| Diagnosis | UMLS:ICD10CM:I50 | Heart failure |
| Diagnosis | UMLS:ICD10CM:I46 | Cardiac arrest |
| Demographics | Deceased | Deceased |
| **ESKD necessitating dialysis** | | |
| Procedure | UMLS:CPT:1012740 | Dialysis Services and Procedures |
| Procedure | UMLS:CPT:90945 | Dialysis procedure other than hemodialysis (eg, peritoneal dialysis, hemofiltration, or other continuous renal replacement therapies), with single evaluation by a physician or other qualified health care professional |
| Diagnosis | UMLS:ICD10CM:N18.6 | End stage renal disease |
| **Secondary outcomes** | | |
| **All-cause mortality** | | |
| Demographics | Deceased | Deceased |
| **Acute myocardial infarction** | | |
| Diagnosis | UMLS:ICD10CM:I21 | Acute myocardial infarction |
| **Heart failure** | | |
| Diagnosis | UMLS:ICD10CM:I50 | Heart failure |
| **Stroke** | | |
| Diagnosis | UMLS:ICD10CM:I61 | Nontraumatic intracerebral hemorrhage |
| Diagnosis | UMLS:ICD10CM:I62 | Other and unspecified nontraumatic intracranial hemorrhage |
| Diagnosis | UMLS:ICD10CM:I63 | Cerebral infarction |
| **Acute kidney injury** | | |
| Diagnosis | UMLS:ICD10CM:N17 | Acute kidney failure |
| **Sensitivity analysis** | | |
| **ESKD, alternative definition** | | |
| Diagnosis | UMLS:ICD10CM:N18.6 | End stage renal disease |
| Diagnosis | UMLS:ICD10CM:Z99.2 | Dependence on renal dialysis |
| **Safety outcomes** | | |
| **Rhabdomyolysis** | | |
| Diagnosis | UMLS:ICD10CM:M62.82 | Rhabdomyolysis |
| **Impaired liver function** | | |
| Diagnosis | UMLS:ICD10CM:K72 | Hepatic failure, not elsewhere classified |
| Diagnosis | UMLS:ICD10CM:R74.8 | Abnormal levels of other serum enzymes |
| Diagnosis | UMLS:ICD10CM:K71.2 | Toxic liver disease with acute hepatitis |
| Diagnosis | UMLS:ICD10CM:R94.5 | Abnormal results of liver function studies |
| **Myopathy** | | |
| Diagnosis | UMLS:ICD10CM:G72.0 | Drug-induced myopathy |
| Diagnosis | UMLS:ICD10CM:M62.82 | Rhabdomyolysis |
| **Functional diarrhea** | | |
| Diagnosis | UMLS:ICD10CM:K59.1 | Functional diarrhea |

## 3. Codes for the baseline covariates for propensity score matching

| **Demographics** | | | |
| --- | --- | --- | --- |
| **Code** | **Name** | | |
| AI | Age at index | | |
| M | Male | | |
| 2106-3 | White | | |
| 2054-5 | Black or African American | | |
| 2028-9 | Asian | | |
| 2131-1 | Other race | | |
| **Diagnosis** | | | |
| **ICD-10-CM code** | **Name** | | |
| F17 | Nicotine dependence | | |
| F41 | Other anxiety disorders | | |
| I20-I25 | Ischemic heart diseases | | |
| I50 | Heart failure | | |
| I60-I69 | Cerebrovascular diseases | | |
| J44 | Other chronic obstructive pulmonary disease | | |
| E00-E07 | Disorders of thyroid gland | | |
| E83 | Disorders of mineral metabolism | | |
| G60-G65 | Polyneuropathies and other disorders of the peripheral nervous system | | |
| I10-I1A | Hypertensive diseases | | |
| I42 | Cardiomyopathy | | |
| I48 | Atrial fibrillation and flutter | | |
| I49 | Other cardiac arrhythmias | | |
| J40-J4A | Chronic lower respiratory diseases | | |
| F02 | Dementia in other diseases classified elsewhere | | |
| Z72.0 | Tobacco use | | |
| F10 | Alcohol related disorders | | |
| E08-E13 | Diabetes mellitus | | |
| M10 | Gout | | |
| Z55-Z65 | Persons with potential health hazards related to socioeconomic and psychosocial circumstances | | |
| Z69-Z76 | Persons encountering health services in other circumstances | | |
| Z77-Z99 | Persons with potential health hazards related to family and personal history and certain conditions influencing health status | | |
| **Procedure** | | | |
| **CPT code (TriNetX curated)** | **Name** | | |
| 1013626 | Office or Other Outpatient Services | | |
| 1013711 | Emergency Department Services | | |
| 1013659 | Hospital Inpatient and Observation Care Services | | |
| 1013829 | Preventive Medicine Services | | |
| **Medication** | | | |
| **Code (ATC, VA class, or RxNorm)** | **Name** | | |
| HS501 | Insulin | | |
| HS502 | Oral hypoglycemic agents, oral | | |
| CV300 | Antiarrhythmics | | |
| B01 | Antithrombotic agents | | |
| C09 | Agents acting on the renin–angiotensin system | | |
| C03 | Diuretics | | |
| C07 | Beta blocking agents | | |
| C08 | Calcium channel blockers | | |
| C04 | Peripheral vasodilators | | |
| C03DA | Aldosterone antagonists | | |
| M01A | Anti-inflammatory and antirheumatic products, non-steroids | | |
| 73689 | Febuxostat | | |
| 519 | Allopurinol | | |
| H03 | Thyroid therapy | | |
| A02BC | Proton pump inhibitors | | |
| **Laboratory data** | | | |
| **TriNetX curated code** | **Name (unit)** | **Data missing rate after matching** | |
|  |  | **Statin + fenofibrate** | **Statin monotherapy** |
| 9004 | Triglyceride in Serum (mg/dL) | 8.0% | 7.7% |
| 9002 | Cholesterol in LDL in Serum (mg/dL) | 16.9% | 15.6% |
| 8001 | Glomerular filtration rate/1.73 sq M.predicted in Serum, by Creatinine-based formula (MDRD) (mL·min⁻¹·1.73 m⁻²) | 2.2% | 1.6% |
| 9083 | Body mass index (kg/m²) | 33.7% | 33.1% |

# **Method S2. The details of propensity score matching in the TriNetX platform**

**(https://support.trinetx.com/hc/en-us/articles/360011978033)**

To conduct PSM using the TNX Research platform, you must first identify two cohorts of interest, index events, outcomes of interest, and attributes of patients which may act as confounders to the outcomes of interest. For the purpose of this article, these attributes will be called “covariates.”

Within Balance, when you run a propensity score matching analysis, the system conducts a propensity score matching to balance the cohorts:

1. For each patient in each cohort, the system computes values for each covariate.
2. These data form a matrix of covariate values for each
3. The system performs a logistic regression on the pooled matrices, to “predict” which cohort each patient originates from. The value of this model for a patient is that patient’s predicted probability of being in the second cohort or “propensity score.”
4. For each patient in the smaller cohort, the system chooses as match from the larger cohort (if any patients in the larger cohort are close enough). The pairs then form a subset of each cohort.

Within Outcomes, when you run compare cohorts after matching, the system conducts a propensity score matching to compare outcomes between the balance the cohorts:

1. For each patient in each cohort, the system computes both the outcome(s) of interest and values for each covariate.
2. These data form a matrix of covariate values for each
3. The system performs a logistic regression on the pooled matrices, to “predict” which cohort each patient originates from. The value of this model for a patient is that patient’s predicted probability of being in the second cohort or “propensity score.”
4. For each patient in the smaller cohort, the system chooses as match from the larger cohort (if any patients in the larger cohort are close enough). The pairs then form a subset of each cohort.
5. The system compares outcomes on these after matching subsets, rather than the original cohorts.

**Values in the Covariate Matrix**

In the covariate matrix, each row represents one patient, and each column represents one covariate. Each cell contains exactly one non-null numerical value.

 All covariates are one of the following forms:

- **Binary:** yes/no;
- **Categorical:** real values placed in categories based on their value; or
- **Continuous:** real values represented directly.

When a covariate is binary (for example, a patient having history of asthma), the cell for that patient and covariate is set to 0 for “not present” or 1 for “is present.”

When a covariate is categorical, the categories are specified as ranges that the continuous variable can take (for example, having a value for a Sodium lab between 130 and 140). Each category becomes a distinct covariate which is either 0 for “not present” or 1 for “is present.” It is possible for all columns to be 0 (for example if there are no values in the time window, or if all values are outside all the categories). It is also possible for multiple values to be 1 (for example if there are multiple values in the time window, or if the categories overlap).

When a covariate is continuous (for example, current age, or age at time of the index event), then the value for that patient (if any) is placed in the relevant cell.

**Details on Scoring and Logistic Regression**

When you run propensity score matching, the system generates a propensity score for each patient in each cohort. The propensity score ranges between 0 and 1 and indicates the predicted probability a patient is in cohort B given the patient’s covariates.

To uses logistic regression to generate the propensity scores, through an implementation of the well-tested, standard software package [scikit-learn](https://scikit-learn.org/). The code used in production is as follows (some logging, etc. removed for clarity):

import numpy as np

from sklearn.linear_model import LogisticRegression

from sklearn.preprocessing import Imputer

from impute import fill_nans

def fill_nans(data, axis=0):

  imp = Imputer(missing_values='NaN', strategy=method, axis=axis)

    return imp.fit_transform(data)

def propensity_scores(matrix_a, matrix_b):

matrix_a = fill_nans(matrix_a, method='mean')

matrix_b = fill_nans(matrix_b, method='mean')

full_matrix = np.concatenate((matrix_a, matrix_b), axis=0)

\# cohort A is "zero" and cohort B is "one" from the regression’s perspective

target_a = np.zeros(matrix_a.shape[0], dtype=float)

target_b = np.ones(matrix_b.shape[0], dtype=float)

target = np.concatenate([target_a, target_b])

lr = LogisticRegression(C=1000)

lr.fit(full_matrix, target)

def get_scores(X):

\# .predict_proba returns [prob_class_zero, prob_class_one] for each row,

\# while the score we want is the probability of being in class B

full_scores = lr.predict_proba(X)

return full_scores[:, 1]

return (get_scores(matrix_a), get_scores(matrix_b))

Here the “score” for each patient is the regression model applied to that patient’s row. Informally, this is the probability that the patient belongs to cohort B, based on the cohorts it has seen.

Here “matrix_a” is the covariate matrix for the cohort you selected as Cohort A. Likewise, “matrix_b” is the covariate matrix for the cohort you selected as Cohort B.  Data are pooled across all HCOs, so all patients in the analysis are a row in one of these matrices. Note that the code instructs the system to replace all missing (NaN) values in each matrix with the mean from that column; however, at time of writing all covariates are either binary, categorical (which expands to a set of binary columns), or continuous but guaranteed to exist, so this imputation is vacuous.

The call “LogisticRegression(C=1000)” means we predict the probability of being in cohort B using logistic regression. The value of C means we weight the mean residual by a factor of 1000 as compared to the norm of the coefficients. This means we are using a very small amount of L2 (ridge) regression, which is needed to make the objective function convex (so the regression converges), but with such a small value, it has very little effect on the model. Due to the very small amount of regularization and the scale of our covariates being constrained, it is not necessary to perform mean normalization as a preprocessing step.

**Matching Details**

Once the system has generated a propensity score for each patient, the system performs matching to identify the matched subsets. We use “greedy nearest neighbor matching” with a caliper of 0.1 pooled standard deviations. Sample code is here:

import numpy as np

def _pooled_sd(floats_a, floats_b):

var_a = np.var(floats_a)

var_b = np.var(floats_b)

return sqrt((var_a + var_b) / 2.0)

def _naive_nearest_neighbor_match(scores_a, scores_b, caliper):

\# Perform nearest neighbor matching. Assume that len(scores_a) <=len(scores_b)

num_a, num_b = len(scores_a), len(scores_b)

max_diff = _pooled_sd(scores_a, scores_b) * caliper

chosen_a = np.zeros(num_a, dtype=bool) # True if the patient is matched, start false

chosen_b = np.zeros(num_b, dtype=bool)

for a_ind in range(0, num_a):

best_diff = max_diff

best_b_ind = None

found_any = False

for b_ind in range(0, num_b):

if chosen_b[b_ind]:

continue

diff = abs(scores_a[a_ind] - scores_b[b_ind])

if diff < best_diff or (diff == best_diff and not found_any):

best_diff = diff

best_b_ind = b_ind

found_any = True

if best_b_ind is not None:

chosen_a[a_ind] = True

chosen_b[best_b_ind] = True

return (chosen_a, chosen_b)

*Note:* the code used in production is highly optimized and significantly longer. The above code is taken directly from our test fixtures and is used to ensure that the optimized code produces identical results to the above simplified code.

We use a “caliper” of 0.1 pooled standard deviations of the propensity scores in aggregate, which means that patients with very different propensity scores are not matched. The system matches patients using the following algorithm:

1. For each patient in cohort A (assumed to be the smaller of the two), and identify the patient whose score is closest to the patient in cohort A from the patients in cohort B who have not yet been matched.
2. If a match is found, mark both as “chosen” and move on.
3. At the end, return the labels of each cohort, indicating which patients were chosen and which were not. The patients who were chosen form the “matched” cohorts.

**Pooling the Cohorts and Compensating for Unbalanced Cohorts across HCOs**

TriNetX pulls data from a federated data network made up of many healthcare organizations across the world. Each site computes a covariate matrix for the patients they contribute to the analysis and sends it to a central processing point to be pooled and analyzed as a single matrix.

The order of the rows in the matrix should not impact the propensity scores generated for each patient; logistic regression is highly stable with respect to permuting the rows.  In contrast, nearest neighbor matching can be influenced by the order of rows in the matrix. For example, if two identical patients in cohort B match equally well to a patient in cohort A, the first will be chosen and the second will not.  Therefore, if the order of the rows carries some information (perhaps as an artefact of the pooling), the order can introduce bias.

To eliminate this bias, we randomize the order of the records in the covariate matrix.  We sort the provider results by the unique ID of the HCO (so the information of “which HCO responded first” is deleted), concatenate the matrices into one large matrix, then shuffle them using np.random.shuffle(full_matrix_a) and np.random.shuffle(full_matrix_b). To assure determinism, a call to np.random.seed(FIXED_SEED) precedes all calls to shuffle, so that successive runs do not change unless the underlying data change.

# **Figure S1. Schematic of study design**

# **Figure S2. Distribution of follow-up time in the propensity score–matched cohort**

Follow-up was truncated at 4 years (1,460 days) after the index date; the last bar comprises patients who completed 4 years of follow-up.

# **Figure S3. Kaplan–Meier curves for secondary outcomes in the propensity score–matched cohort**

(A) All-cause mortality, (B) acute myocardial infarction, (C) heart failure, (D) stroke, and (E) acute kidney injury. The grey area marks the first 3 months after the index date; follow-up began at this landmark, and patients who died during this interval were excluded. Coloured bands indicate 95% confidence intervals; P values are from log-rank tests. Numbers at risk, back-calculated from the Kaplan–Meier estimates exported by TriNetX, are shown below each panel.

**Table S1.** Target trial emulation

| **Target trial component** | **Target Trial (Hypothetical RCT)** | **Target Trial Emulation (This Study)** |
| --- | --- | --- |
| **Clinical question** | Among adults with non-dialysis-dependent CKD, ischemic heart disease, dyslipidemia, and hypertriglyceridemia who are initiating statin therapy, does initiating fenofibrate together with statin therapy reduce cardiorenal events compared with statin monotherapy? | Among adults in the TriNetX network with CKD, ischemic heart disease, dyslipidemia, hypertriglyceridemia, and statin initiation, the study compared outcomes between patients who started fenofibrate within 3 months of statin initiation and those who did not. |
| **Eligibility criteria** | Adults aged ≥18 years with non-dialysis-dependent CKD, eGFR ≤60 mL/min/1.73 m², ischemic heart disease, dyslipidemia, triglycerides ≥150 mg/dL, and an indication for statin therapy. Patients with dialysis-dependent kidney disease, recent statin or fenofibrate use, active liver disease, malignancy, or other contraindications to fenofibrate would be excluded. | Adults aged ≥18 years with ≥3 visits, dyslipidemia, eGFR ≤60 mL/min/1.73 m², triglycerides ≥150 mg/dL, ischemic heart disease within 1 year before the index date, and statin use after meeting eligibility criteria were identified.  Patients were excluded for statin or fenofibrate use within 1 year before the index date, neoplasm or liver disease before the index date, dialysis within 6 months before the index date, or death within 3 months after the index date. |
| **Treatment strategies** | Strategy 1: initiate statin plus fenofibrate combination therapy. Strategy 2: initiate statin monotherapy without fenofibrate. Treatment would be assigned at baseline and continued according to protocol unless clinically contraindicated. | Patients were classified as combination-therapy users if fenofibrate was prescribed within 3 months of statin initiation and as statin-monotherapy users if it was not. |
| **Assignment procedure** | Eligible patients would be randomly assigned to statin–fibrate combination therapy or statin monotherapy, ensuring exchangeability of measured and unmeasured baseline characteristics. | Treatment assignment was observational. To approximate randomization, 1:1 propensity-score matching was performed using demographic, clinical, health care utilization, medication, and laboratory covariates measured before the index date. |
| **Grace period** | Not applicable; the assigned strategy would start at randomization. | 3 months after statin initiation; fenofibrate prescribed during this period defined the combination strategy. |
| **Time zero** | The date of randomization, coinciding with confirmation of eligibility and initiation of the assigned treatment strategy. | Statin initiation (index date), when eligibility and baseline covariates were assessed; outcome follow-up started at the end of the grace period (3-month landmark). |
| **Baseline covariate assessment** | Baseline characteristics would be measured before randomization, including demographics, comorbidities, medication use, health care utilization, and laboratory values. | Baseline covariates were assessed during the 1 year before the index date and included age, sex, BMI, race, socioeconomic and lifestyle factors, comorbidities, medication use, health care utilization, eGFR, triglycerides, and LDL cholesterol. |
| **Follow-up period** | Patients would be followed from randomization until outcome occurrence, death, loss to follow-up, treatment discontinuation if relevant to the estimand, or administrative end of follow-up. | From the end of the grace period (3-month landmark) until the outcome, death or last recorded encounter (censoring for non-fatal outcomes), or 4 years after the index date. |
| **Primary outcomes** | Major adverse cardiovascular events and ESKD necessitating dialysis would be prespecified as primary or co-primary outcomes. | Expanded MACE (acute myocardial infarction, stroke, heart failure, cardiac arrest, or all-cause death) and ESKD necessitating dialysis. |
| **Secondary outcomes** | Mortality, acute myocardial infarction, heart failure, stroke, acute kidney injury, and prespecified safety outcomes including rhabdomyolysis, liver dysfunction, myopathy, and diarrhea. | Secondary outcomes included mortality, acute myocardial infarction, heart failure, stroke, acute kidney injury, and safety outcomes including rhabdomyolysis, impaired liver function, myopathy, and functional diarrhea. |
| **Causal contrast / estimand** | Intention-to-treat effect of assignment to statin–fibrate combination therapy versus statin monotherapy. A per-protocol effect could be estimated separately if adherence and treatment changes were protocolized. | Observational analogue of the intention-to-treat effect of initiating combination therapy versus statin monotherapy, among patients who survived the grace period. |
| **Statistical analysis** | Primary analysis would compare randomized groups using time-to-event models, Kaplan–Meier curves, absolute risk differences, NNT, and RMST/RMSTD, with prespecified subgroup and safety analyses. | Cox proportional hazards models, Kaplan–Meier curves, log-rank tests, propensity-score matching, ARR, events prevented per 1,000 patients, NNT, RMSTD, subgroup analyses, negative control outcomes, E-values, and landmark/sensitivity analyses were used. |
| **Control of confounding** | Randomization would control measured and unmeasured confounding in expectation. | Propensity-score matching was used to balance measured baseline covariates.  Residual confounding from unmeasured factors such as adherence, frailty, clinician prescribing preferences, diet, and dialysis-initiation thresholds remains possible. |
| **Protocol deviations / adherence** | Adherence, discontinuation, treatment switching, dose changes, and adverse-event–driven withdrawal would be prospectively captured. | No adherence rules were applied; patients were analysed according to the strategy initiated during the grace period. Prescription records do not capture dispensing, adherence, dose, or discontinuation. |
| **Safety monitoring** | Safety outcomes would be actively monitored through protocolized laboratory testing, symptom assessment, and adverse-event reporting. | Safety outcomes were assessed using EHR-coded diagnoses and available clinical data, which may undercapture mild or subclinical adverse events. |
| **Interpretation** | A well-conducted RCT would support causal inference regarding the efficacy and safety of adding fibrate therapy to statin therapy in the target population. | The emulated design supports stronger causal interpretation than a conventional retrospective cohort, but findings should remain framed as associations because treatment was not randomized and residual confounding and misclassification cannot be excluded. |

**Abbreviations:** RCT, randomized controlled trial; CKD, chronic kidney disease; TriNetX, TriNetX research network; eGFR, estimated glomerular filtration rate; MACE, major adverse cardiovascular events; ESKD, end-stage kidney disease; AMI, acute myocardial infarction; AKI, acute kidney injury; ARR, absolute risk reduction; NNT, number needed to treat; RMSTD, restricted mean survival time difference.

**Table S2.** STROBE statement

| **Section/Topic** | **Item No.** | **Recommendation** | **Location in manuscript** |
| --- | --- | --- | --- |
| Title and abstract | 1 | (a) Indicate the study’s design with a commonly used term in the title or the abstract | Abstract: Methods |
|  |  | (b) Provide in the abstract an informative and balanced summary of what was done and what was found | Abstract |
| **Introduction** | | | |
| Background/rationale | 2 | Explain the scientific background and rationale for the investigation being reported | Introduction, paragraphs 1–4 |
| Objectives | 3 | State specific objectives, including any prespecified hypotheses | Introduction, paragraph 4 |
| **Methods** | | | |
| Study design | 4 | Present key elements of study design early in the paper | Abstract: Methods  Methods: Study design and cohort, paragraphs 1–2  Supplementary Table S1  Supplementary Figure S1 |
| Setting | 5 | Describe the setting, locations, and relevant dates, including periods of recruitment, exposure, follow-up, and data collection | Methods: Data source, paragraph 1  Methods: Study design and cohort, paragraph 1  Methods: Study outcomes, paragraph 1 |
| Participants | 6 | (a) Give the eligibility criteria, and the sources and methods of selection of participants. Describe methods of follow-up | Methods: Study design and cohort, paragraphs 1–2  Methods: Study outcomes, paragraph 1  Figure 1  Supplementary Method S1 |
|  |  | (b) For matched studies, give matching criteria and number of exposed and unexposed | Methods: Baseline covariates and PSM, paragraphs 1–2  Results: Baseline characteristics, paragraphs 1–2  Table 1 |
| Variables | 7 | Clearly define all outcomes, exposures, predictors, potential confounders, and effect modifiers. Give diagnostic criteria, if applicable | Methods: Study design and cohort, paragraphs 1–2  Methods: Baseline covariates and PSM, paragraph 1  Methods: Study outcomes, paragraph 1  Methods: Statistical analysis, paragraph 3  Supplementary Method S1 |
| Data sources/measurement | 8* | For each variable of interest, give sources of data and details of methods of assessment (measurement). Describe comparability of assessment methods if there is more than one group | Methods: Data source, paragraph 1  Methods: Study design and cohort, paragraph 1  Methods: Baseline covariates and PSM, paragraphs 1–2  Supplementary Methods S1 and S2 |
| Bias | 9 | Describe any efforts to address potential sources of bias | Methods: Study design and cohort, paragraphs 1–2  Methods: Baseline covariates and PSM, paragraph 2  Methods: Sensitivity analysis, paragraph 1 |
| Study size | 10 | Explain how the study size was arrived at | Methods: Study design and cohort, paragraphs 1–2  Figure 1 |
| Quantitative variables | 11 | Explain how quantitative variables were handled in the analyses. If applicable, describe which groupings were chosen and why | Methods: Baseline covariates and PSM, paragraph 2  Methods: Statistical analysis, paragraph 1  Table 1  Figures 3 and 4 |
| Statistical methods | 12 | (a) Describe all statistical methods, including those used to control for confounding | Methods: Baseline covariates and PSM, paragraph 2  Methods: Statistical analysis, paragraphs 1, 2, and 4  Supplementary Method S2 |
|  |  | (b) Describe any methods used to examine subgroups and interactions | Methods: Statistical analysis, paragraph 3 |
|  |  | (c) Explain how missing data were addressed | Methods: Baseline covariates and PSM, paragraphs 1–2  Methods: Statistical analysis, paragraph 4 |
|  |  | (d) If applicable, explain how loss to follow-up was addressed | Methods: Study outcomes, paragraph 1 |
|  |  | (e) Describe any sensitivity analyses | Methods: Sensitivity analysis, paragraph 1 |
| **Results** | | | |
| Participants | 13* | (a) Report numbers of individuals at each stage of study—eg numbers potentially eligible, examined for eligibility, confirmed eligible, included in the study, completing follow-up, and analysed | Methods: Study design and cohort, paragraphs 1–2  Results: Baseline characteristics, paragraphs 1–2  Figure 1 |
|  |  | (b) Give reasons for non-participation at each stage | Methods: Study design and cohort, paragraph 1  Figure 1 |
|  |  | (c) Consider use of a flow diagram | Figure 1 |
| Descriptive data | 14* | (a) Give characteristics of study participants (eg demographic, clinical, social) and information on exposures and potential confounders | Results: Baseline characteristics, paragraphs 1–2  Table 1 |
|  |  | (b) Indicate number of participants with missing data for each variable of interest | Table 1, footnote  Supplementary Method S1, section 3 |
|  |  | (c) Summarise follow-up time (eg, average and total amount) | Results: Baseline characteristics, paragraph 2  Supplementary Figure S2 |
| Outcome data | 15* | Report numbers of outcome events or summary measures over time | Results: Cardiorenal outcomes, paragraphs 1–2  Table 2  Figure 2  Supplementary Figure S3  Supplementary Table S3 |
| Main results | 16 | (a) Give unadjusted estimates and, if applicable, confounder-adjusted estimates and their precision (eg, 95% confidence interval). Make clear which confounders were adjusted for and why they were included | Results: Cardiorenal outcomes, paragraph 2  Table 2  Methods: Baseline covariates and PSM, paragraph 1  Supplementary Table S5 |
|  |  | (b) Report category boundaries when continuous variables were categorized | Table 1  Figures 3 and 4  Supplementary Table S6 |
|  |  | (c) If relevant, consider translating estimates of relative risk into absolute risk for a meaningful time period | Results: Clinical application, paragraphs 1–2  Supplementary Table S3 |
| Other analyses | 17 | Report other analyses done—eg analyses of subgroups and interactions, and sensitivity analyses | Results: Results of subgroup analysis, paragraph 1  Results: Results of sensitivity analysis, paragraph 1  Results: Safety profile, paragraph 1  Figures 3 and 4  Supplementary Tables S4–S10 |
| **Discussion** | | | |
| Key results | 18 | Summarise key results with reference to study objectives | Discussion, paragraph 1 |
| Limitations | 19 | Discuss limitations of the study, taking into account sources of potential bias or imprecision. Discuss both direction and magnitude of any potential bias | Discussion, paragraphs 6–7 |
| Interpretation | 20 | Give a cautious overall interpretation of results considering objectives, limitations, multiplicity of analyses, results from similar studies, and other relevant evidence | Discussion, paragraphs 2–5 and 8 |
| Generalisability | 21 | Discuss the generalisability (external validity) of the study results | Discussion, paragraphs 5 and 7 |
| **Other information** | | | |
| Funding | 22 | Give the source of funding and the role of the funders for the present study and, if applicable, for the original study on which the present article is based | Funding |

*Give information separately for exposed and unexposed groups.

Items are those of the STROBE checklist for cohort studies. Locations refer to the revised main manuscript: section and subsection headings as printed, with paragraphs numbered from the start of each subsection (or of the section where it has no subsections).

**Table S3.** Annual absolute risk reduction and clinical impact

| **Outcome / Metric** | **Statin + fenofibrate versus statin monotherapy** | | | |
| --- | --- | --- | --- | --- |
|  | **Year 1** | **Year 2** | **Year 3** | **Year 4** |
| **Expanded MACE** |  |  |  |  |
| ARR, % (95% CI)^a^ | 3.07 (0.53–5.61) | 2.88 (0.14–5.61) | 2.14 (−0.73 to 5.01) | 2.23 (−0.78 to 5.25) |
| Events prevented per 1,000 patients | 30.7 | 28.8 | 21.4 | 22.3 |
| NNT (95% CI)^b^ | 33 (18–188) | 35 (18–702) | 47 (NS) | 45 (NS) |
| RMSTD, days (95% CI)^c^ | 5.64 (0.24–11.05) | 16.61 (2.60–30.62) | 26.00 (3.18–48.82) | 34.30 (2.59–66.01) |
| **ESKD necessitating dialysis** |  |  |  |  |
| ARR, % (95% CI)^a^ | 2.81 (1.50–4.12) | 3.17 (1.56–4.79) | 1.93 (0.08–3.77) | 1.86 (−0.27 to 3.99) |
| Events prevented per 1,000 patients | 28.1 | 31.7 | 19.3 | 18.6 |
| NNT (95% CI)^b^ | 36 (25–67) | 32 (21–65) | 52 (27–1,195) | 54 (NS) |
| RMSTD, days (95% CI)^c^ | 5.94 (3.18–8.71) | 17.74 (10.12–25.37) | 27.17 (13.94–40.40) | 33.12 (13.62–52.63) |

^a^Absolute risk reduction; a positive value indicates a lower risk with combination therapy.

^b^Number needed to treat, calculated as 1/ARR and rounded up to the next whole patient; NS, not statistically significant.

^c^Restricted mean survival time difference, the average event-free time gained up to each year.

Abbreviations: CI, confidence interval; ESKD, end-stage kidney disease; MACE, major adverse cardiovascular events.

**Table S4.** Falsification test

| **Outcome** | **After matching** | | | | | |
| --- | --- | --- | --- | --- | --- | --- |
|  | **Statin + fenofibrate**  **(n = 3,295)** | | **Statin monotherapy**  **(n = 3,295)** | | **Statin + fenofibrate vs. statin monotherapy** | |
|  | **Events, n** | **Risk, %** | **Events, n** | **Risk, %** | **HR (95% CI)** | **P value** |
| Accidental falls | 396 | 12.0 | 398 | 12.1 | 1.05 (0.91–1.20) | 0.513 |
| Pressure ulcer | 131 | 4.0 | 135 | 4.1 | 1.01 (0.80–1.29) | 0.904 |
| Screening colonoscopy^a^ | 349 | 10.6 | 371 | 11.3 | 0.98 (0.84–1.13) | 0.764 |

Negative control outcomes were analyzed in a separately matched cohort with the same cohort definitions (3,295 pairs) and assessed from 3 months to 4 years after the index date: accidental falls (ICD-10-CM W00–W19), pressure ulcer (L89), and screening colonoscopy (Z12.11, encounter for screening for malignant neoplasm of colon).

HRs were estimated with Cox proportional hazards models and P values with log-rank tests.

^a^The proportional-hazards assumption was not met for this outcome (Schoenfeld test, P = 0.033); the HR is an average over follow-up.

Abbreviations: CI, confidence interval; HR, hazard ratio.

**Table S5.** Propensity score matching models

| **Outcome** | **After matching, statin + fenofibrate versus statin monotherapy** | | | | | |
| --- | --- | --- | --- | --- | --- | --- |
|  | **Model 1** | | **Model 2** | | **Model 3** | |
|  | **HR (95% CI)** | **P value** | **HR (95% CI)** | **P value** | **HR (95% CI)** | **P value** |
| Expanded MACE^#^ | 0.85 (0.79–0.91) | <0.001 | 0.95 (0.89–1.03) | 0.213 | 0.97 (0.90–1.04) | 0.333 |
| All-cause mortality | 1.08 (0.93–1.24) | 0.326 | 1.02 (0.88–1.18) | 0.777 | 0.98 (0.85–1.13) | 0.787 |
| Acute myocardial infarction | 0.90 (0.81–1.02) | 0.087 | 0.95 (0.84–1.07) | 0.399 | 1.03 (0.91–1.16) | 0.675 |
| Heart failure | 0.85 (0.78–0.92) | <0.001 | 0.97 (0.89–1.06) | 0.507 | 0.94 (0.87–1.03) | 0.163 |
| Stroke | 0.98 (0.84–1.14) | 0.771 | 0.95 (0.81–1.12) | 0.562 | 0.97 (0.83–1.14) | 0.736 |
| Acute kidney injury | 1.07 (0.97–1.18) | 0.188 | 1.09 (0.99–1.21) | 0.085 | 1.00 (0.91–1.11) | 0.947 |
| ESKD necessitating dialysis | 0.77 (0.65–0.90) | 0.001 | 0.81 (0.69–0.96) | 0.015 | 0.74 (0.63–0.87) | <0.001 |

Model 1 was matched for age, sex, race, body mass index, lifestyle factors, socioeconomic status, and health-care utilization; Model 2 additionally for baseline comorbidities; and Model 3 additionally for baseline medication use.

^#^Expanded MACE included acute myocardial infarction, stroke, heart failure, cardiac arrest, and all-cause death.

Abbreviations: CI, confidence interval; ESKD, end-stage kidney disease; HR, hazard ratio; MACE, major adverse cardiovascular events.

**Table S6.** Baseline characteristics with the extended covariate set

| **Characteristic** | **Before matching** | | | **After matching** | | |
| --- | --- | --- | --- | --- | --- | --- |
|  | **Statin +**  **fenofibrate**  **(n = 3,658)** | **Statin**  **monotherapy**  **(n = 66,082)** | **SMD** | **Statin +**  **fenofibrate**  **(n = 3,644)** | **Statin**  **monotherapy**  **(n = 3,644)** | **SMD** |
| **Demographic characteristics** |  |  |  |  |  |  |
| Age, years, mean ± SD | 66.4 ± 10.3 | 67.6 ± 10.7 | 0.116 | 66.5 ± 10.3 | 66.4 ± 10.7 | 0.004 |
| Male sex, n (%) | 2,526 (69.1) | 38,141 (57.7) | 0.237 | 2,513 (69.0) | 2,485 (68.2) | 0.017 |
| Race, n (%) |  |  |  |  |  |  |
| White | 2,619 (71.6) | 45,933 (69.5) | 0.046 | 2,617 (71.8) | 2,648 (72.7) | 0.019 |
| Black or African American | 131 (3.6) | 6,843 (10.4) | 0.268 | 131 (3.6) | 125 (3.4) | 0.009 |
| Asian | 115 (3.1) | 2,387 (3.6) | 0.026 | 115 (3.2) | 131 (3.6) | 0.024 |
| Other | 83 (2.3) | 1,738 (2.6) | 0.023 | 83 (2.3) | 77 (2.1) | 0.011 |
| Body mass index, kg/m², mean ± SD | 32.1 ± 6.6 | 31.3 ± 6.8 | 0.117 | 32.1 ± 6.6 | 31.9 ± 6.5 | 0.022 |
| ≤25, n (%)^a^ | 397 (10.9) | 10,385 (15.7) | 0.144 | 397 (10.9) | 399 (10.9) | 0.002 |
| 25–30, n (%)^a^ | 1,000 (27.3) | 21,010 (31.8) | 0.098 | 1,000 (27.4) | 993 (27.3) | 0.004 |
| 30–35, n (%)^a^ | 1,066 (29.1) | 20,060 (30.4) | 0.027 | 1,066 (29.3) | 1,090 (29.9) | 0.014 |
| ≥35, n (%)^a^ | 878 (24.0) | 15,918 (24.1) | 0.002 | 878 (24.1) | 904 (24.8) | 0.017 |
| **Socioeconomic and lifestyle factors, n (%)** |  |  |  |  |  |  |
| Socioeconomic and psychosocial circumstances (Z55–Z65) | 81 (2.2) | 1,909 (2.9) | 0.043 | 81 (2.2) | 80 (2.2) | 0.002 |
| Health services in other circumstances (Z69–Z76) | 494 (13.5) | 10,239 (15.5) | 0.057 | 494 (13.6) | 489 (13.4) | 0.004 |
| Family and personal history hazards (Z77–Z99) | 2,704 (73.9) | 50,174 (75.9) | 0.046 | 2,700 (74.1) | 2,714 (74.5) | 0.009 |
| Tobacco use | 180 (4.9) | 3,590 (5.4) | 0.023 | 180 (4.9) | 186 (5.1) | 0.008 |
| Nicotine dependence | 586 (16.0) | 10,841 (16.4) | 0.010 | 585 (16.1) | 618 (17.0) | 0.024 |
| Alcohol-related disorders | 85 (2.3) | 1,724 (2.6) | 0.018 | 85 (2.3) | 99 (2.7) | 0.024 |
| **Health-care utilization, n (%)** |  |  |  |  |  |  |
| Outpatient services | 1,419 (38.8) | 29,353 (44.4) | 0.114 | 1,418 (38.9) | 1,405 (38.6) | 0.007 |
| Inpatient and observation care | 1,369 (37.4) | 24,402 (36.9) | 0.010 | 1,368 (37.5) | 1,401 (38.4) | 0.019 |
| Emergency department services | 1,363 (37.3) | 25,613 (38.8) | 0.031 | 1,362 (37.4) | 1,372 (37.7) | 0.006 |
| Preventive medicine services | 129 (3.5) | 2,333 (3.5) | <0.001 | 129 (3.5) | 132 (3.6) | 0.004 |
| **Comorbidities, n (%)** |  |  |  |  |  |  |
| Hypertensive diseases | 3,351 (91.6) | 60,035 (90.8) | 0.027 | 3,337 (91.6) | 3,350 (91.9) | 0.013 |
| Diabetes mellitus | 2,785 (76.1) | 40,179 (60.8) | 0.335 | 2,771 (76.0) | 2,777 (76.2) | 0.004 |
| Heart failure | 1,219 (33.3) | 23,729 (35.9) | 0.054 | 1,218 (33.4) | 1,266 (34.7) | 0.028 |
| Atrial fibrillation and flutter | 714 (19.5) | 13,811 (20.9) | 0.034 | 713 (19.6) | 733 (20.1) | 0.014 |
| Other cardiac arrhythmias | 414 (11.3) | 8,901 (13.5) | 0.065 | 414 (11.4) | 389 (10.7) | 0.022 |
| Cardiomyopathy | 285 (7.8) | 6,325 (9.6) | 0.063 | 285 (7.8) | 307 (8.4) | 0.022 |
| Cerebrovascular diseases | 716 (19.6) | 14,260 (21.6) | 0.050 | 716 (19.6) | 761 (20.9) | 0.031 |
| Chronic lower respiratory diseases | 901 (24.6) | 15,738 (23.8) | 0.019 | 901 (24.7) | 895 (24.6) | 0.004 |
| Chronic obstructive pulmonary disease | 681 (18.6) | 10,922 (16.5) | 0.055 | 681 (18.7) | 667 (18.3) | 0.010 |
| Disorders of thyroid gland | 765 (20.9) | 12,745 (19.3) | 0.041 | 762 (20.9) | 768 (21.1) | 0.004 |
| Disorders of mineral metabolism | 396 (10.8) | 7,752 (11.7) | 0.029 | 396 (10.9) | 399 (10.9) | 0.003 |
| Gout | 397 (10.9) | 6,556 (9.9) | 0.031 | 391 (10.7) | 370 (10.2) | 0.019 |
| Anxiety disorders | 676 (18.5) | 11,324 (17.1) | 0.035 | 671 (18.4) | 670 (18.4) | 0.001 |
| Polyneuropathy | 184 (5.0) | 3,508 (5.3) | 0.013 | 183 (5.0) | 199 (5.5) | 0.020 |
| Dementia | 45 (1.2) | 817 (1.2) | 0.001 | 45 (1.2) | 54 (1.5) | 0.021 |
| Acute kidney injury^a^ | 1,144 (31.3) | 19,228 (29.1) | 0.047 | 1,143 (31.4) | 1,156 (31.7) | 0.008 |
| **Medications, n (%)** |  |  |  |  |  |  |
| Antithrombotic agents | 3,136 (85.7) | 52,970 (80.2) | 0.149 | 3,124 (85.7) | 3,114 (85.5) | 0.008 |
| Beta-blockers | 2,891 (79.0) | 47,845 (72.4) | 0.155 | 2,878 (79.0) | 2,890 (79.3) | 0.008 |
| Renin–angiotensin system blockers | 2,216 (60.6) | 34,765 (52.6) | 0.161 | 2,204 (60.5) | 2,262 (62.1) | 0.033 |
| Insulin | 2,060 (56.3) | 29,588 (44.8) | 0.232 | 2,051 (56.3) | 2,069 (56.8) | 0.010 |
| Diuretics | 1,771 (48.4) | 30,728 (46.5) | 0.038 | 1,764 (48.4) | 1,753 (48.1) | 0.006 |
| Calcium channel blockers | 1,760 (48.1) | 29,320 (44.4) | 0.075 | 1,751 (48.1) | 1,739 (47.7) | 0.007 |
| Proton pump inhibitors | 1,487 (40.7) | 24,838 (37.6) | 0.063 | 1,484 (40.7) | 1,491 (40.9) | 0.004 |
| Antiarrhythmics | 1,497 (40.9) | 28,688 (43.4) | 0.050 | 1,496 (41.1) | 1,508 (41.4) | 0.007 |
| Glucose-lowering drug classes^a^ |  |  |  |  |  |  |
| SGLT2 inhibitors | 464 (12.7) | 5,030 (7.6) | 0.169 | 459 (12.6) | 434 (11.9) | 0.021 |
| GLP-1 receptor agonists | 286 (7.8) | 3,164 (4.8) | 0.125 | 283 (7.8) | 288 (7.9) | 0.005 |
| DPP-4 inhibitors | 419 (11.5) | 4,132 (6.3) | 0.184 | 412 (11.3) | 432 (11.9) | 0.017 |
| Biguanides | 856 (23.4) | 9,453 (14.3) | 0.234 | 844 (23.2) | 834 (22.9) | 0.007 |
| Sulfonylureas | 527 (14.4) | 5,911 (8.9) | 0.171 | 520 (14.3) | 517 (14.2) | 0.002 |
| Alpha-glucosidase inhibitors | 64 (1.7) | 279 (0.4) | 0.128 | 58 (1.6) | 58 (1.6) | <0.001 |
| Thiazolidinediones | 124 (3.4) | 882 (1.3) | 0.136 | 122 (3.3) | 117 (3.2) | 0.008 |
| Thyroid therapy | 675 (18.5) | 9,321 (14.1) | 0.118 | 670 (18.4) | 672 (18.4) | 0.001 |
| Nonsteroidal anti-inflammatory drugs | 585 (16.0) | 10,845 (16.4) | 0.011 | 583 (16.0) | 568 (15.6) | 0.011 |
| Aldosterone antagonists | 320 (8.7) | 5,151 (7.8) | 0.035 | 316 (8.7) | 337 (9.2) | 0.020 |
| Allopurinol | 289 (7.9) | 4,775 (7.2) | 0.026 | 289 (7.9) | 282 (7.7) | 0.007 |
| Peripheral vasodilators | 211 (5.8) | 1,898 (2.9) | 0.143 | 203 (5.6) | 190 (5.2) | 0.016 |
| Febuxostat | 100 (2.7) | 1,142 (1.7) | 0.068 | 97 (2.7) | 95 (2.6) | 0.003 |
| **Laboratory values and blood pressure** |  |  |  |  |  |  |
| eGFR, mL/min/1.73 m², mean ± SD | 47.4 ± 21.2 | 48.0 ± 23.1 | 0.025 | 47.5 ± 21.2 | 47.8 ± 22.3 | 0.018 |
| 30–60, n (%)^a^ | 2,933 (80.2) | 49,351 (74.7) | 0.132 | 2,919 (80.1) | 2,965 (81.4) | 0.032 |
| 15–30, n (%)^a^ | 862 (23.6) | 13,869 (21.0) | 0.062 | 859 (23.6) | 903 (24.8) | 0.028 |
| ≤15, n (%)^a^ | 501 (13.7) | 10,840 (16.4) | 0.076 | 495 (13.6) | 478 (13.1) | 0.014 |
| Triglycerides, mg/dL, mean ± SD | 310.5 ± 269.5 | 222.2 ± 131.2 | 0.417 | 309.6 ± 269.4 | 287.0 ± 207.6 | 0.094 |
| ≥500, n (%) | 491 (13.4) | 2,167 (3.3) | 0.373 | 479 (13.1) | 462 (12.7) | 0.014 |
| LDL cholesterol, mg/dL, mean ± SD | 82.1 ± 39.4 | 92.5 ± 45.4 | 0.245 | 82.2 ± 39.5 | 82.8 ± 39.2 | 0.016 |
| ≥190, n (%) | 64 (1.7) | 2,230 (3.4) | 0.103 | 64 (1.8) | 64 (1.8) | <0.001 |
| HbA1c, %, mean ± SD^a^ | 7.6 ± 2.0 | 7.3 ± 2.0 | 0.175 | 7.6 ± 2.0 | 7.7 ± 2.1 | 0.043 |
| ≤6, n (%)^a^ | 648 (17.7) | 15,638 (23.7) | 0.147 | 648 (17.8) | 635 (17.4) | 0.009 |
| 6–8, n (%)^a^ | 1,571 (42.9) | 23,922 (36.2) | 0.138 | 1,561 (42.8) | 1,547 (42.5) | 0.008 |
| ≥8, n (%)^a^ | 1,082 (29.6) | 14,506 (22.0) | 0.175 | 1,070 (29.4) | 1,124 (30.8) | 0.032 |
| Systolic blood pressure, mmHg, mean ± SD^a^ | 129.9 ± 21.2 | 130.4 ± 21.8 | 0.021 | 129.9 ± 21.2 | 129.4 ± 21.9 | 0.026 |
| Diastolic blood pressure, mmHg, mean ± SD^a^ | 70.0 ± 12.8 | 71.0 ± 13.1 | 0.076 | 70.0 ± 12.8 | 70.8 ± 12.7 | 0.060 |

Values are n (%) or mean ± SD. An SMD <0.1 indicates adequate balance between groups; after matching, all SMDs were <0.1.

^a^Added to the propensity score model of the primary analysis (Table 1); the seven glucose-lowering drug classes replaced oral hypoglycemic agents. Codes: SGLT2 inhibitors, ATC A10BK; GLP-1 receptor agonists, A10BJ; DPP-4 inhibitors, A10BH; biguanides, A10BA; sulfonylureas, A10BB; alpha-glucosidase inhibitors, A10BF; thiazolidinediones, A10BG; acute kidney injury, ICD-10-CM N17.

Laboratory values, blood pressure, and body mass index are the mean of all measurements in the year before the index date among patients with at least one measurement (after matching: eGFR, 97.6% and 97.7%; triglycerides, 91.8% and 90.5%; LDL cholesterol, 83.4% and 83.2%; HbA1c, 76.6% and 76.2%; systolic blood pressure, 74.2% and 74.5%; diastolic blood pressure, 74.2% and 74.4%; body mass index, 69.8% and 70.6%). Categories are based on all patients in each group; a patient with measurements in more than one range is counted in each. The other triglyceride and LDL cholesterol categories of the model are not shown (SMD after matching ≤0.035).

Abbreviations: DPP-4, dipeptidyl peptidase-4; eGFR, estimated glomerular filtration rate; GLP-1, glucagon-like peptide-1; HbA1c, glycated hemoglobin; LDL, low-density lipoprotein; SD, standard deviation; SGLT2, sodium–glucose cotransporter 2; SMD, standardized mean difference.

**Table S7.** Clinical outcomes with the extended covariate set

| **Outcome** | **Statin + fenofibrate**  **(n = 3,644)**  **Events, n (%)** | **Statin monotherapy**  **(n = 3,644)**  **Events, n (%)** | **HR (95% CI)** | **P value** |
| --- | --- | --- | --- | --- |
| Expanded MACE^#^ | 1,795 (49.3) | 1,948 (53.5) | 0.89 (0.84–0.95) | <0.001 |
| All-cause mortality | 513 (14.1) | 524 (14.4) | 1.01 (0.89–1.13) | 0.957 |
| Acute myocardial infarction | 671 (18.4) | 732 (20.1) | 0.93 (0.83–1.03) | 0.148 |
| Heart failure | 1,252 (34.4) | 1,417 (38.9) | 0.87 (0.81–0.94) | <0.001 |
| Stroke | 369 (10.1) | 413 (11.3) | 0.91 (0.79–1.05) | 0.186 |
| Acute kidney injury | 1,009 (27.7) | 1,052 (28.9) | 0.98 (0.90–1.07) | 0.706 |
| ESKD necessitating dialysis | 333 (9.1) | 403 (11.1) | 0.84 (0.72–0.97) | 0.016 |

Patients were matched on the extended covariate set shown in Table S6; outcomes were assessed from 3 months to 4 years after the index date. HRs were estimated with Cox proportional hazards models and P values with log-rank tests. The proportional-hazards assumption was met for all outcomes (Schoenfeld test, all P > 0.05).

^#^Expanded MACE included acute myocardial infarction, stroke, heart failure, cardiac arrest, and all-cause death.

Abbreviations: CI, confidence interval; ESKD, end-stage kidney disease; HR, hazard ratio; MACE, major adverse cardiovascular events.

**Table S8.** Hazard ratios over cumulative follow-up windows starting at the 3-month landmark

| **Outcome** | **After matching, statin + fenofibrate versus statin monotherapy** | | | | | | | |
| --- | --- | --- | --- | --- | --- | --- | --- | --- |
|  | **3 months to 1 year** | | **3 months to 2 years** | | **3 months to 3 years** | | **3 months to 5 years** | |
|  | **HR (95% CI)** | **P value** | **HR (95% CI)** | **P value** | **HR (95% CI)** | **P value** | **HR (95% CI)** | **P value** |
| Expanded MACE^#^ | 0.93 (0.85–1.01) | 0.101 | 0.91 (0.84–0.99) | 0.023 | 0.92 (0.85–0.99) | 0.032 | 0.93 (0.86–0.99) | 0.035 |
| All-cause mortality | 1.13 (0.87–1.47) | 0.361 | 1.07 (0.88–1.30) | 0.505 | 1.03 (0.87–1.23) | 0.711 | 1.03 (0.90–1.18) | 0.678 |
| Acute myocardial infarction | 0.93 (0.79–1.09) | 0.372 | 1.00 (0.87–1.15) | 0.988 | 0.92 (0.81–1.05) | 0.237 | 0.99 (0.88–1.12) | 0.896 |
| Heart failure | 0.97 (0.87–1.08) | 0.566 | 0.90 (0.82–0.99) | 0.030 | 0.95 (0.87–1.04) | 0.273 | 0.94 (0.86–1.02) | 0.122 |
| Stroke | 1.00 (0.80–1.23) | 0.969 | 1.00 (0.83–1.20) | 0.979 | 0.95 (0.80–1.13) | 0.591 | 0.89 (0.76–1.04) | 0.135 |
| Acute kidney injury | 0.98 (0.85–1.13) | 0.762 | 1.01 (0.90–1.13) | 0.871 | 1.07 (0.96–1.20) | 0.207 | 1.04 (0.94–1.14) | 0.465 |
| ESKD necessitating dialysis | 0.73 (0.59–0.90) | 0.003 | 0.77 (0.64–0.93) | 0.006 | 0.77 (0.64–0.91) | 0.003 | 0.77 (0.66–0.90) | 0.001 |

^#^Expanded MACE included acute myocardial infarction, stroke, heart failure, cardiac arrest, and all-cause death.

Abbreviations: CI, confidence interval; ESKD, end-stage kidney disease; HR, hazard ratio; MACE, major adverse cardiovascular events.

**Table S9.** Clinical outcomes with combination therapy defined as same-day co-prescription (follow-up from 1 day to 4 years after the index date)

| **Outcome** | **After matching** | | | | | | |
| --- | --- | --- | --- | --- | --- | --- | --- |
|  | **Statin + fenofibrate**  **(n = 2,175)** | | **Statin monotherapy**  **(n = 2,175)** | | **Statin + fenofibrate vs. statin monotherapy** | | |
|  | **Events, n** | **Risk, %** | **Events, n** | **Risk, %** | **HR (95% CI)** | **P value** | **E-value** |
| Expanded MACE^#^ | 1,157 | 53.2 | 1,312 | 60.3 | 0.84 (0.78–0.91) | <0.001 | 1.67 (1.43) |
| All-cause mortality | 317 | 14.6 | 316 | 14.5 | 1.05 (0.90–1.23) | 0.525 | 1.28 (1.00) |
| Acute myocardial infarction | 444 | 20.4 | 515 | 23.7 | 0.86 (0.76–0.98) | 0.023 | 1.60 (1.16) |
| Heart failure | 802 | 36.9 | 899 | 41.3 | 0.89 (0.81–0.97) | 0.012 | 1.50 (1.21) |
| Stroke | 241 | 11.1 | 275 | 12.6 | 0.89 (0.75–1.06) | 0.200 | 1.50 (1.00) |
| Acute kidney injury | 668 | 30.7 | 714 | 32.8 | 0.95 (0.86–1.06) | 0.381 | 1.29 (1.00) |
| ESKD necessitating dialysis | 205 | 9.4 | 277 | 12.7 | 0.74 (0.62–0.89) | 0.001 | 2.04 (1.50) |

Combination therapy was defined as fenofibrate prescribed on the same day as the first statin prescription; the comparator group comprised statin initiators without fenofibrate on that day. Follow-up ran from the day after the index date to 4 years, without exclusion of early deaths, in a separately matched cohort.

^#^Expanded MACE included acute myocardial infarction, stroke, heart failure, cardiac arrest, and all-cause death.

Abbreviations: CI, confidence interval; ESKD, end-stage kidney disease; HR, hazard ratio; MACE, major adverse cardiovascular events.

**Table S10.** Alternative definition of ESKD and safety outcomes in a separate matched analysis

| **Outcome** | **After matching** | | | | | |
| --- | --- | --- | --- | --- | --- | --- |
|  | **Statin + fenofibrate**  **(n = 3,660)** | | **Statin monotherapy**  **(n = 3,660)** | | **Statin + fenofibrate vs. statin monotherapy** | |
|  | **Events, n** | **Risk, %** | **Events, n** | **Risk, %** | **HR (95% CI)** | **P value** |
| ESKD (N18.6 or Z99.2) | 330 | 9.0 | 408 | 11.1 | 0.80 (0.69–0.93) | 0.003 |
| Rhabdomyolysis | 41 | 1.1 | 31 | 0.8 | 1.34 (0.84–2.14) | 0.217 |
| Impaired liver function | 192 | 5.2 | 209 | 5.7 | 0.92 (0.76–1.12) | 0.417 |
| Myopathy | 44 | 1.2 | 37 | 1.0 | 1.21 (0.78–1.87) | 0.402 |
| Functional diarrhea^*^ | — | — | — | — | — | — |

This analysis was run separately on the TriNetX platform with the cohort definitions, 3-month landmark, and 4-year follow-up window of the primary analysis; its matched sample differs from that of the primary analysis because each analysis is performed independently. P values are from log-rank tests. Codes are listed in Supplementary Method S1.

^*^The number of patients with functional diarrhea was too small for results to be reported by the platform.

Abbreviations: CI, confidence interval; ESKD, end-stage kidney disease; HR, hazard ratio.
